# A new samarium complex of 1,3-bis(pyridin-3-ylmethyl)thiourea on boehmite nanoparticles as a practical and recyclable nanocatalyst for the selective synthesis of tetrazoles

**DOI:** 10.1038/s41598-023-33109-y

**Published:** 2023-04-11

**Authors:** Parisa Moradi, Tavan Kikhavani, Yunes Abbasi Tyula

**Affiliations:** 1 Department of Chemistry, Faculty of Science, Ilam University, P.O. Box 69315516, Ilam, Iran; 2 Department of Chemical Engineering, Faculty of Engineering, Ilam University, Ilam, Iran

**Keywords:** Catalyst synthesis, Heterogeneous catalysis

## Abstract

Boehmite is a natural and environmentally friendly compound. Herein boehmite nanoparticles were primarily synthesized and, then, their surface were modified via 3-choloropropyltrimtoxysilane (CPTMS). Afterwards, a new samarium complex was stabilized on the surface of the modified boehmite nanoparticles (Sm-bis(PYT)@boehmite). The obtained nanoparticles were characterized using thermogravimetric analysis (TGA), energy dispersive X-ray spectroscopy (EDS), Brunauer–Emmett–Teller (BET), wavelength dispersive X-ray spectroscopy (WDX), scanning electron microscope (SEM), Fourier transform infrared spectroscopy (FT-IR), Inductively coupled plasma mass spectrometry (ICP-MS), dynamic light scattering (DLS), and X-ray diffraction (XRD) pattern. Sm-bis(PYT)@boehmite was used as an environmentally friendly, efficient, and organic–inorganic hybrid nanocatalyst in the homoselective synthesis of tetrazoles in polyethylene glycol 400 (PEG-400) as a green solvent. Notably, Sm-bis(PYT)@boehmite is stable and has a heterogeneous nature. Thus, it can be reused for several runs without any re-activation.

## Introduction

Natural materials and the supported transition metals are great candidates for catalytic applications due to their availability, relatively low-cost, bio-degradability, and bio-compatibility^1–9^. Boehmite is one of the attractive natural mineral materials, which was recently employed as support in fabricating organometallic catalysts^1,10,11^. In fact, boehmite is aluminum oxide hydroxide (γ-AlOOH), which is the most stable alumina phase in nature after gibbsite^12–15^. Moreover, boehmite turns into γ-Al_2_O_3_, δ-Al_2_O_3_, θ-Al_2_O_3_, and α-Al_2_O_3_ at temperatures of 450 °C, 900 °C, 1000 °C, and 1200 °C, respectively^16^.

Nowadays, it is accepted that boehmite consists of double layers of octahedron structure from oxygen and a central aluminum atom with a cubic orthorhombic unit cell in which aluminum is surrounded by six oxygen atoms^17–19^. The two layers are connected via hydrogen bonds^20^. Boehmite can be synthesized using various methods such as hydrolysis of aluminum salts^19^, solid-state decomposition of gibbsite^21^, precipitation in an aqueous solution from aluminum salt solutions^22^, sol–gel procedures^23^, hydrothermal procedures^24^ and solvothermal procedures^12^. Most of the boehmite synthetic methods customize the morphology, shape and surface characteristics (pore volume, specific surface area, and pore structure) and also physical and chemical properties. However, boehmite nanomaterials have been rarely used as an ideal support to stabilize various catalysts due to their unique properties such as excellent surface area (> 300 m^2^/g), nanometer size of crystallite, non-toxicity, easy availability, and mechanical and thermal stability (up to 450 °C)^18,25,26^. In this sense, it is worth mentioning that boehmite nanomaterials can be employed as a support to stabilize several transition metals catalysts such as palladium^27^, nickel^28^, platinum^29^, molybdenum^30,31^, vanadium^31^, cobalt^32^, copper^33^, manganese^34^, iron^35^, rhodium^36^, ruthenium^37^ and zirconium^28^. Boehmite nanomaterials have also attracted attention in coatings^38^, absorbents^39^, flame retardant^40^, ceramics^41^, optical materials^42^, vaccine adjuvants^43^, cosmetic products^32^ and in synthesizing alumina as starting materials^44^. However, boehmite nanoparticles have some specific disadvantages (e.g. impurities of nitrate ions), which can affect the surface property and pore crystalline structure. On the other hand, as mentioned above, boehmite nanoparticles may be converted to Al_2_O_3_ at high temperatures; but, as the organic reactions take place at temperatures lower than the boehmite phase change, this cannot effect on the boehmite application in catalysis.

Samarium has rarely been reported as a catalyst for the synthesis of organic compounds. Regarding the fact that selective and reusable catalysts are the main principle of green chemistry, herein a new complex of samarium was investigated using 1,3-bis(pyridin-3-ylmethyl)thiourea on boehmite nanoparticle (Sm-bis(PYT)@boehmite) as a stable, practical, and recyclable nanocatalyst in the homoselective synthesis of 5-substituted 1H-tetrazole derivatives in PEG-400 as a green solvent. Due to the act that one of the principles of green chemistry is to use reusable, cheap and sustainable catalysts, different heterogeneous supported catalysts based on mesoporous materials^45–48^, MOF structures^49^, boehmite^50,51^, carbon materials^52^, polymers^53^, magnetic particles^54–58^ etc. have been reported as catalysts. Moreover, another principle of green chemistry is to apply safe solvents (such as PEG) and safe reagents.

Besides, tetrazole derivatives which are an important group of organo-heterocyclic compounds can be used in various fields such as coordination chemistry, drugs, synthetic organic chemistry, medicinal chemistry as surrogates for carboxylic acids, catalysis technology, the photographic industry, and organometallic chemistry as effective stabilizers of metallopeptidase structures^59–70^. Furthermore, tetrazole derivatives have some specific biological properties, i.e. analgesics, herbicides, anti-proliferative, anti-inflammatory, antimicrobial, anti-HIV, and anticancer properties^18,71–78^.

## Results and discussion

**Figure 1:**
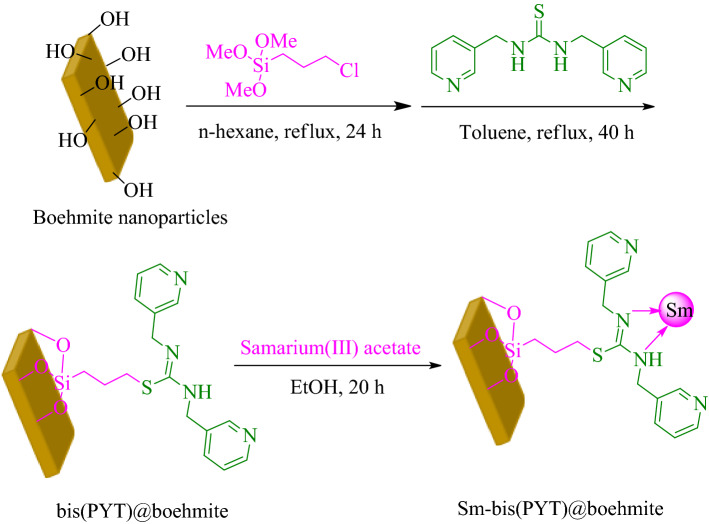
Synthesis of Sm-bis(PYT)@boehmite.

In the first step, modified boehmite nanoparticles through (3-chloropropyl)triethoxysilane were synthesized based on the new procedure^60^. Subsequently, a new complex of samarium was fabricated on their surface (Sm-bis(PYT)@boehmite) (Fig. 1). Sm-bis(PYT)@boehmite was employed as a practical, reusable, and homoselective nanocatalyst used to synthesize tetrazoles. This is the first report proposing the immobilization of 1,3-bis(pyridin-3-ylmethyl)thiourea on boehmite nanoparticles. Moreover, this is the first report in which the samarium complex of 1,3-bis(pyridin-3-ylmethyl)thiourea was used as a catalyst in the synthesis of organic reactions. Therefore, it can be said that this catalyst is an innovation in organic reactions. This nanocatalyst was characterized using N_2_ adsorption–desorption isotherms, TGA, EDS, WDX, SEM, FT-IR, ICP-MS, DLS and XRD techniques.

**Figure 2:**
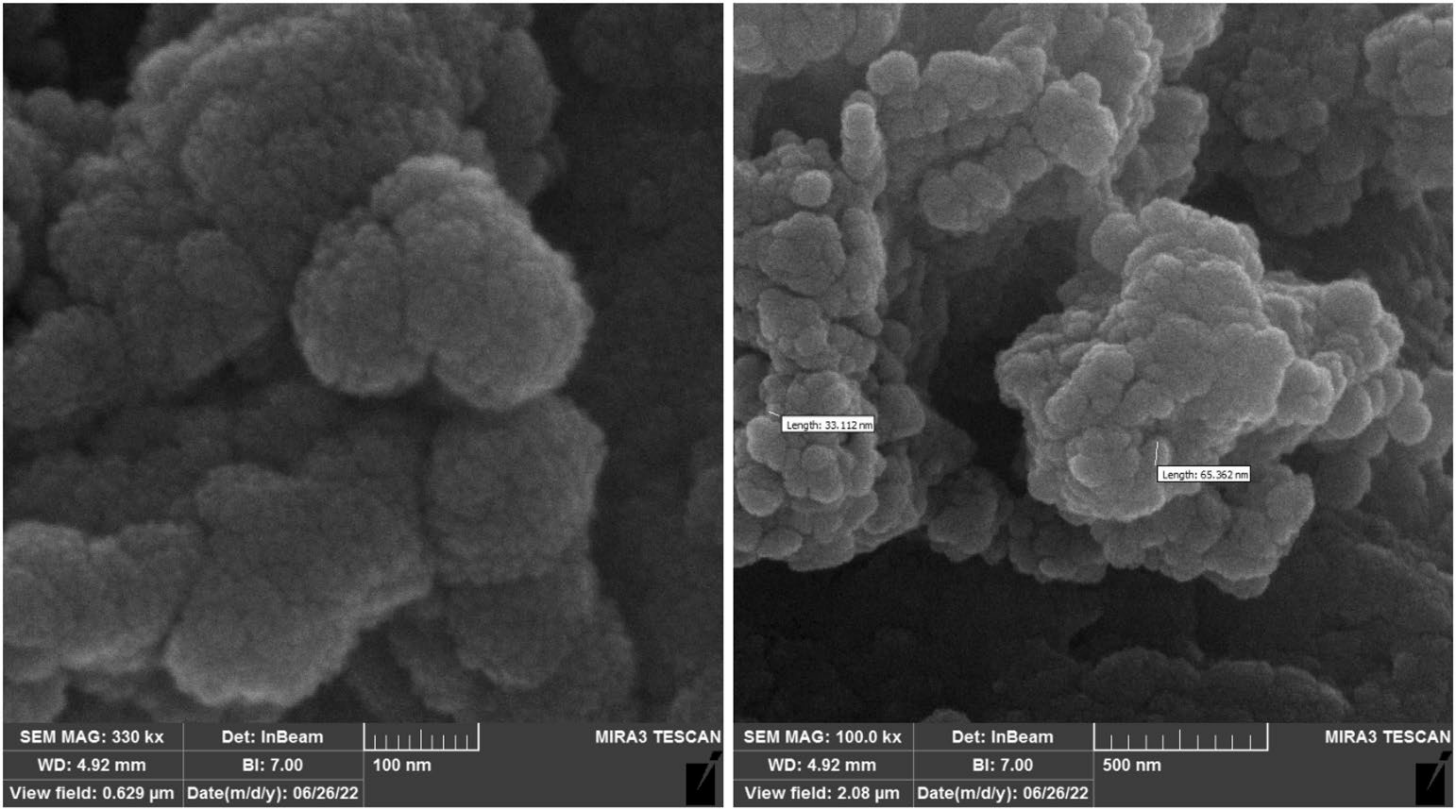
SEM images of Sm-bis(PYT)@boehmite.

**Figure 3:**
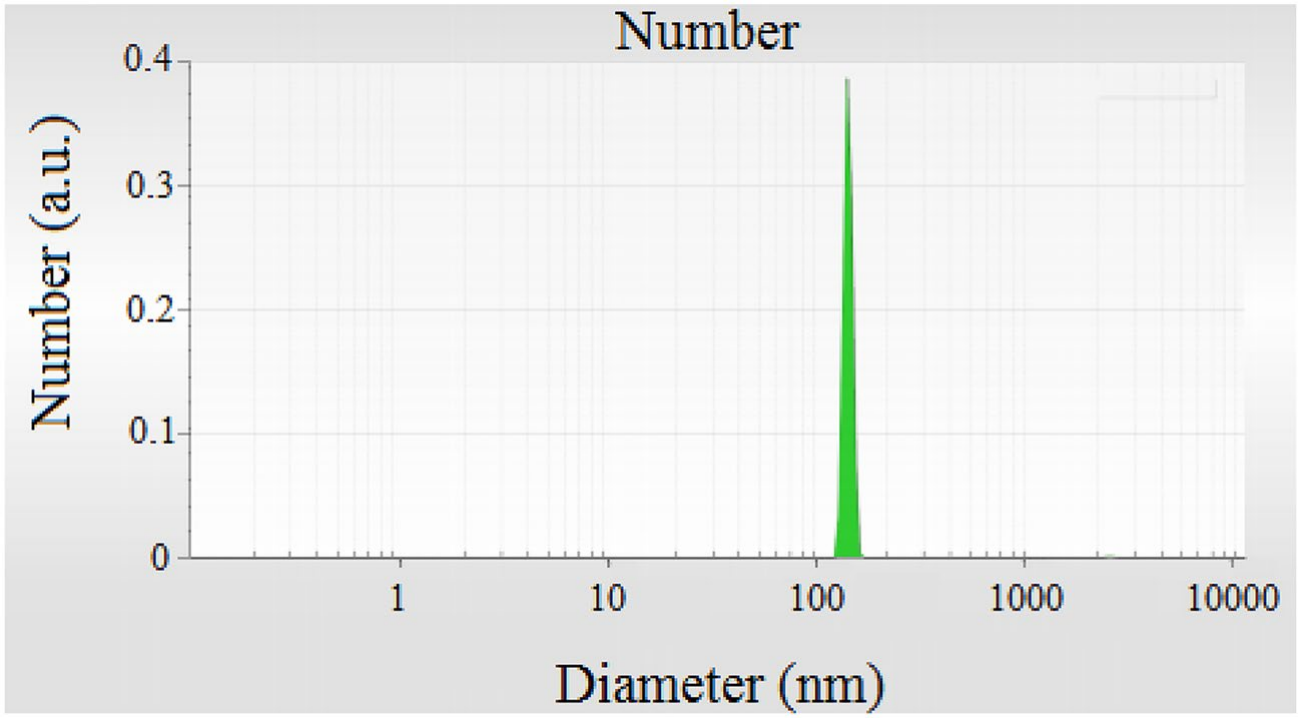
DLS analysis of Sm-bis(PYT)@boehmite.

FESEM-TESCAN MIRA III Scanning Electron Microscope device from Czechia country was employed to study the shape, morphology, and diameters of Sm-bis(PYT)@boehmite. The SEM images are shown in Fig. 2. As shown, the particles of Sm-bis(PYT)@boehmite are formed in uniform spherical shapes and relatively homogeneous diameters of less than 70 nm. Dynamic light scattering (DLS) of Sm-bis(PYT)@boehmite is shown in Fig. 3. Based on DLS analysis, the diameter of Sm-bis(PYT)@boehmite was found to be 129.55 nm, which is greater than the SEM analysis results due to the agglomeration and solvation of the catalyst particles in water^79^.

**Figure 4:**
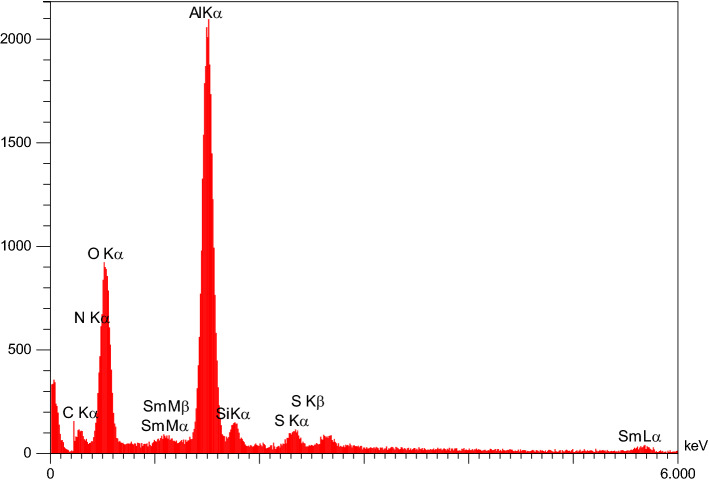
EDS diagram of Sm-bis(PYT)@boehmite.

**Figure 5:**
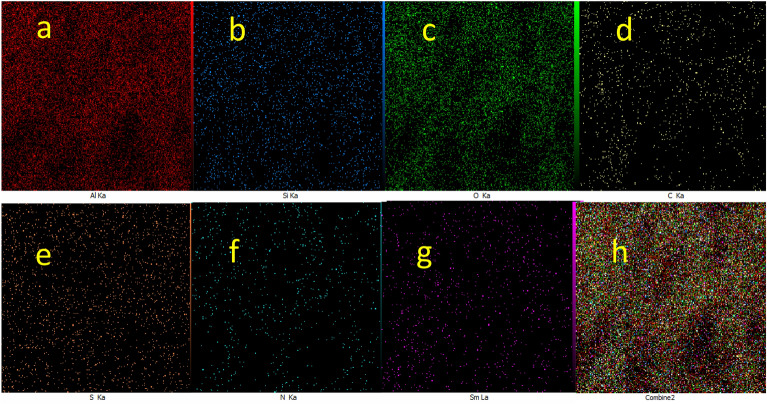
Elemental mapping of (**a**) Al, (**b**) Si, (**c**) O, (**d**) C, (**e**) S, (**f**) N, (**g**) Sm and (**h**) combine of all elements for Sm-bis(PYT)@boehmite.

Regarding the elemental composition of Sm-bis(PYT)@boehmite, the energy-dispersive X-ray spectroscopy (EDS) analysis was used—indicating that Sm-bis(PYT)@boehmite is organized from Al, O, Si, C, N, S, and Sm elements. As shown in Fig. 4, the intensity of aluminum and oxygen peaks is higher than other elements, which form the skeleton of boehmite nanoparticles. Furthermore, the presence of Si, C, N, S, and Sm elements indicates the successful stabilization of the samarium complex on the surface of boehmite nanoparticles. In addition, wavelength dispersive X-ray spectroscopy (WDX) analysis (Fig. 5) illustrates the homogeneous distribution of Al, Si, O, C, N, S, and Sm in the structure of Sm-bis(PYT)@boehmite. Moreover, the exact amount of samarium metal was calculated using ICP-MS analysis. The exact content of samarium—in the structure of Sm-bis(PYT)@boehmite—was obtained as 0.386 mmol/g.

**Figure 6:**
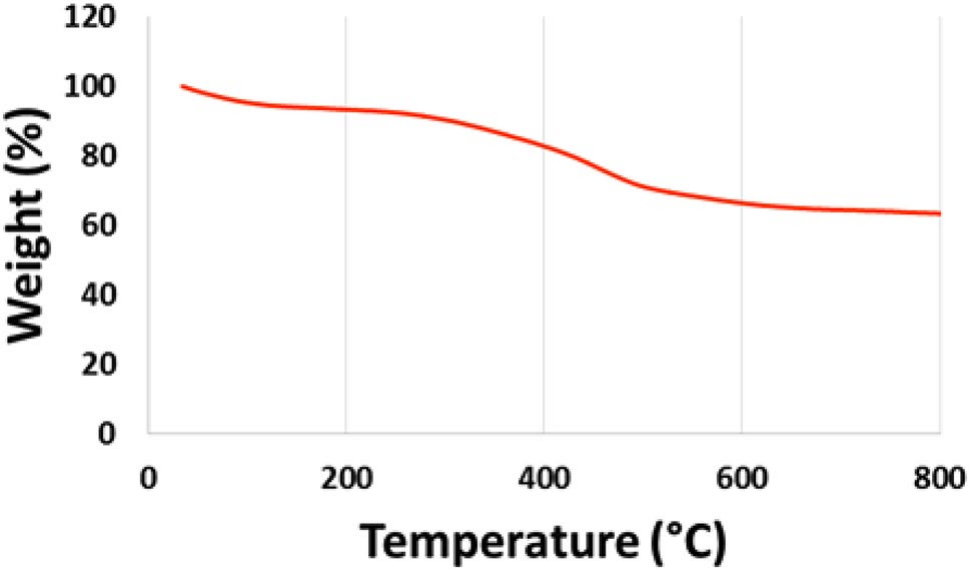
TGA diagram of Sm-bis(PYT)@boehmite.

TGA analysis can be employed to determine the content of inorganic materials and organic compounds in an organic–inorganic hybrid sample and to calculate thermal stability. Therefore, TGA analysis of Sm-bis (PYT)@boehmite was recorded from 25 to 800 °C within an increasing temperature rate of 10 °C/min under air atmosphere (Fig. 6). In this analysis, a small weight loss (7% of weight) up to 150 °C occurred, corresponding to the evaporation of solvents^80,81^. As shown, no weight loss occurred at up to 300 °C except evaporation of solvents, indicating excellent thermal stability of Sm-bis(PYT)@boehmite. Therefore, Sm-bis(PYT)@boehmite can be used as a catalyst under hard conditions in a wide range of organic reactions. TGA analysis of Sm-bis(PYT)@boehmite illustrated a considerable mass loss (27% of weight) between 300 and 600 °C due to the decomposition of immobilized organic layers on the surface of modified boehmite nanoparticles^60^.

**Figure 7:**
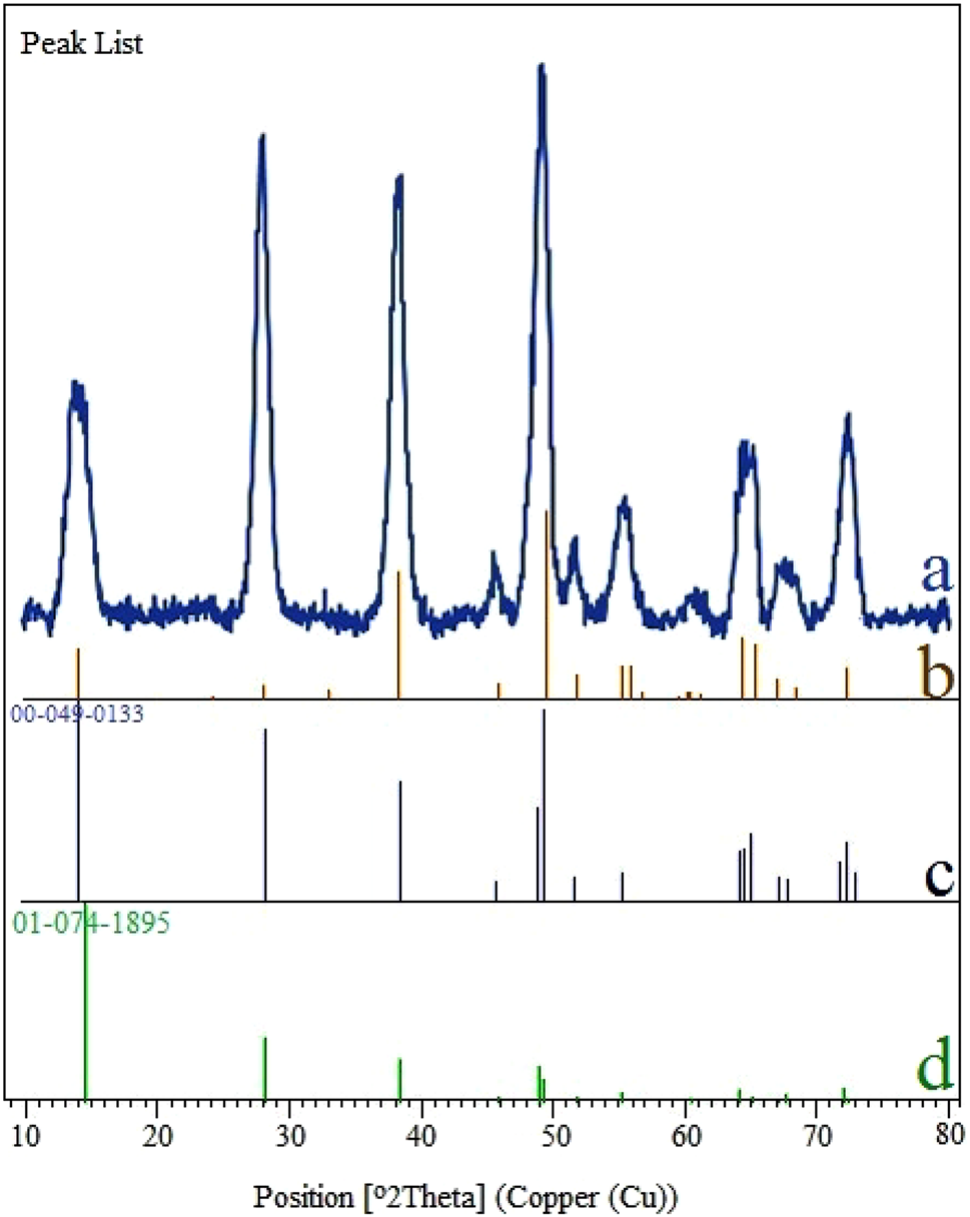
Original XRD pattern of boehmite NPs (**a**), Observed peaks list from normal XRD pattern of boehmite NPs (**b**), standard pattern code 00-049-0133 of boehmite NPs (**c**), and standard pattern code 01-074-1895 of boehmite NPs (**d**).

The X-ray diffraction (XRD) pattern is shown in Fig. 7. The XRD pattern exhibited several peaks of 2θ position at 14.01° (0 2 0), 27.93° (1 2 0), 38.19° (0 3 1), 45.89° (1 3 1), 49.64° (0 5 1), 51.81° (2 0 0), 55.24° (1 5 1), 58.66° (0 8 0), 64.34° (2 3 1), 66.98° (0 0 2), 68.49° (1 7 1), and 72.30° (2 5 1), which can be indexed to the cubic orthorhombic unit cell of boehmite according to X-ray diffraction pattern (Joint Committee on Powder Diffraction Standards (JCPDS)-No. 00-049-0133 and JCPDS-No. 01-074-1895)^82–86^. Moreover, a list of the identified patterns from XRD results of boehmite NPs (AlO(OH)) is summarized in Table 1.

**Table 1:** Identified patterns list from XRD results of boehmite NPs (Aluminum Oxide Hydroxide).

Visible	Ref. code	Score	Compound name	Displacement [°2Th.]	Scale factor	Chemical formula
*	00-049-0133	42	Aluminum oxide hydroxide	0.000	0.683	AlO(OH)
*	01-074-1895	23	Aluminum oxide hydroxide	0.000	0.571	AlO(OH)

**Figure 8:**
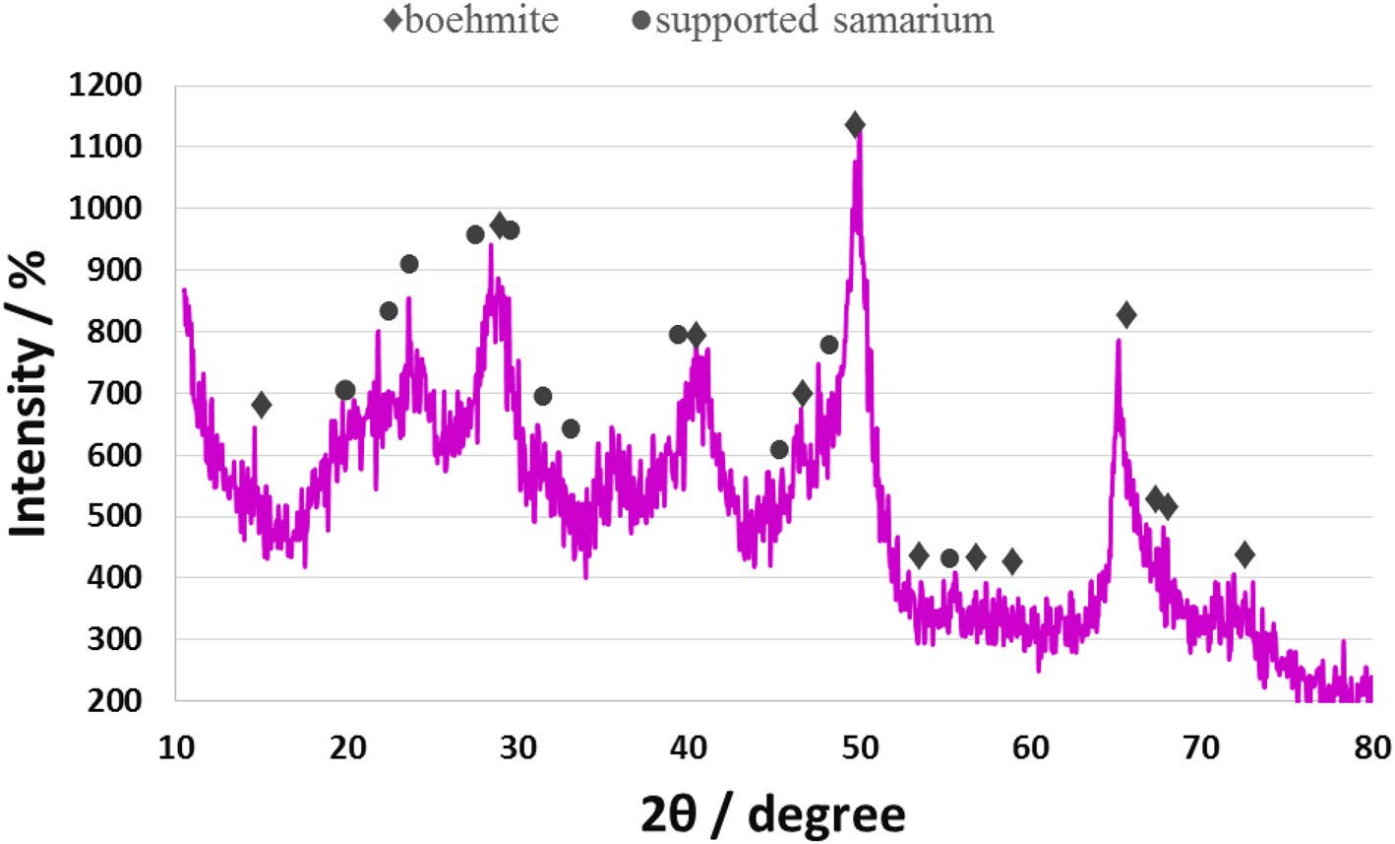
Normal XRD pattern of Sm-bis(PYT)@boehmite.

X-ray diffraction (XRD) pattern of Sm-bis(PYT)@boehmite is presented in Fig. 8. The XRD pattern of Sm-bis(PYT)@boehmite shows several peaks of 2θ position at 14.49° (0 2 0), 28.29° (1 2 0), 38.84° (0 3 1), 46.09° (1 3 1), 49.69° (0 5 1), 52.09° (2 0 0), 56.34° (1 5 1), 58.99° (0 8 0), 64.89° (2 3 1), 66.19° (0 0 2), 67.99° (1 7 1), and 71.99° (2 5 1). These peaks confirm that boehmite nanoparticles are stable as the orthorhombic unit cell (according to XRD pattern codes JCPDS-No. 00-049-0133 and JCPDS-No. 01-074-1895)^12,60^ after modification or stabilization of Sm-complex. Moreover, several peaks of 2θ value at 19.09°, 21.59°, 27.59°, 28.94°, 30.84°, 32.04°, 39.44°, 45.24°, 47.64°, and 55.54° positions are related to the samarium on boehmite nanoparticles^87^. In addition, a broad peak of 2θ from 16° to 25° is related to the amorphous SiO_2_^88^.

**Figure 9:**
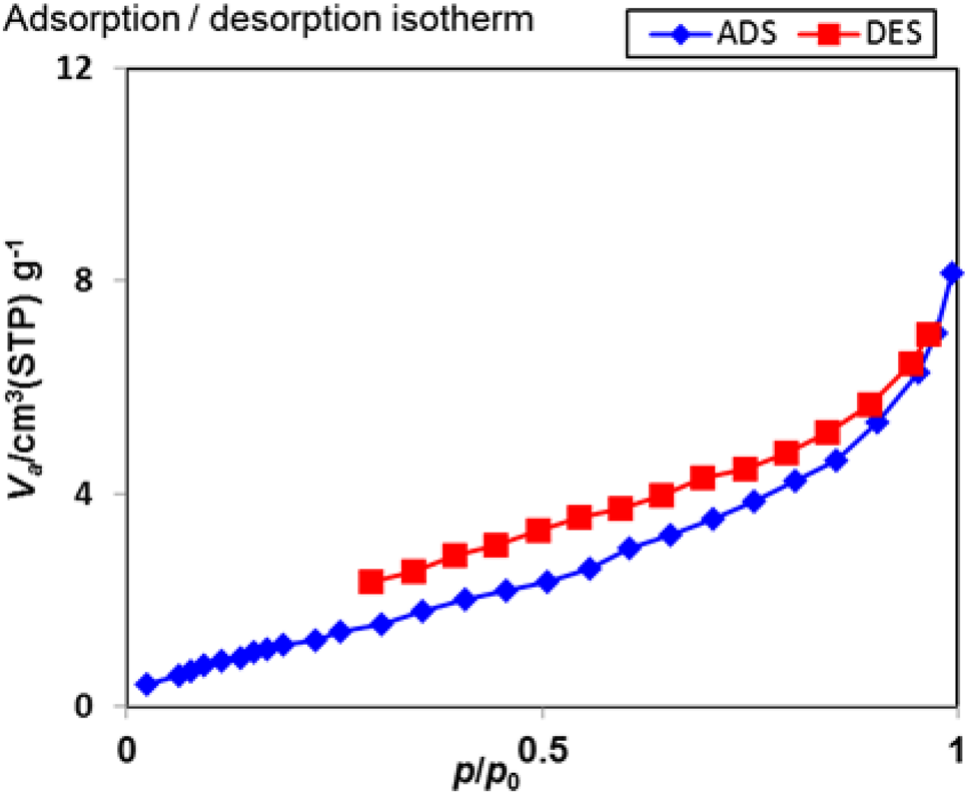
N_2_ adsorption–desorption isotherms of Sm-bis(PYT)@boehmite.

The N_2_ adsorption–desorption isotherms of Sm-bis(PYT)@boehmite are shown in Fig. 9. Moreover, the results of the BET analysis are summarized in Table 2. Based on Brunauer–Emmett–Teller (BET), the surface area of Sm-bis(PYT)@boehmite is 5.30 m^2^/g, which is lower than the unmodified boehmite nanoparticles (which reported about 128.8 m^2^/g^19^). In this sense, it is worth mentioning that, the pore volume of Sm-bis(PYT)@boehmite was calculated at 0.012 cm^3^/g, which is lower than the unmodified boehmite nanoparticles (reported at about 0.22 cm^3^/g^19^). As observed, BET surface area and pore volume of Sm-bis(PYT)@boehmite are lower than the unmodified boehmite nanoparticles due to the grafting of Sm-complex on boehmite nanoparticles.

**Table 2:** Textural properties of boehmite nanoparticles and Sm-bis(PYT)@boehmite.

Entry	Sample	S_BET_ (m^2^/g)	Pore volume (cm^3^/g)
1	Boehmite nanoparticles	128.8^19^	0.22^19^
2	Sm-bis(PYT)@boehmite	5.30	0.012

**Figure 10:**
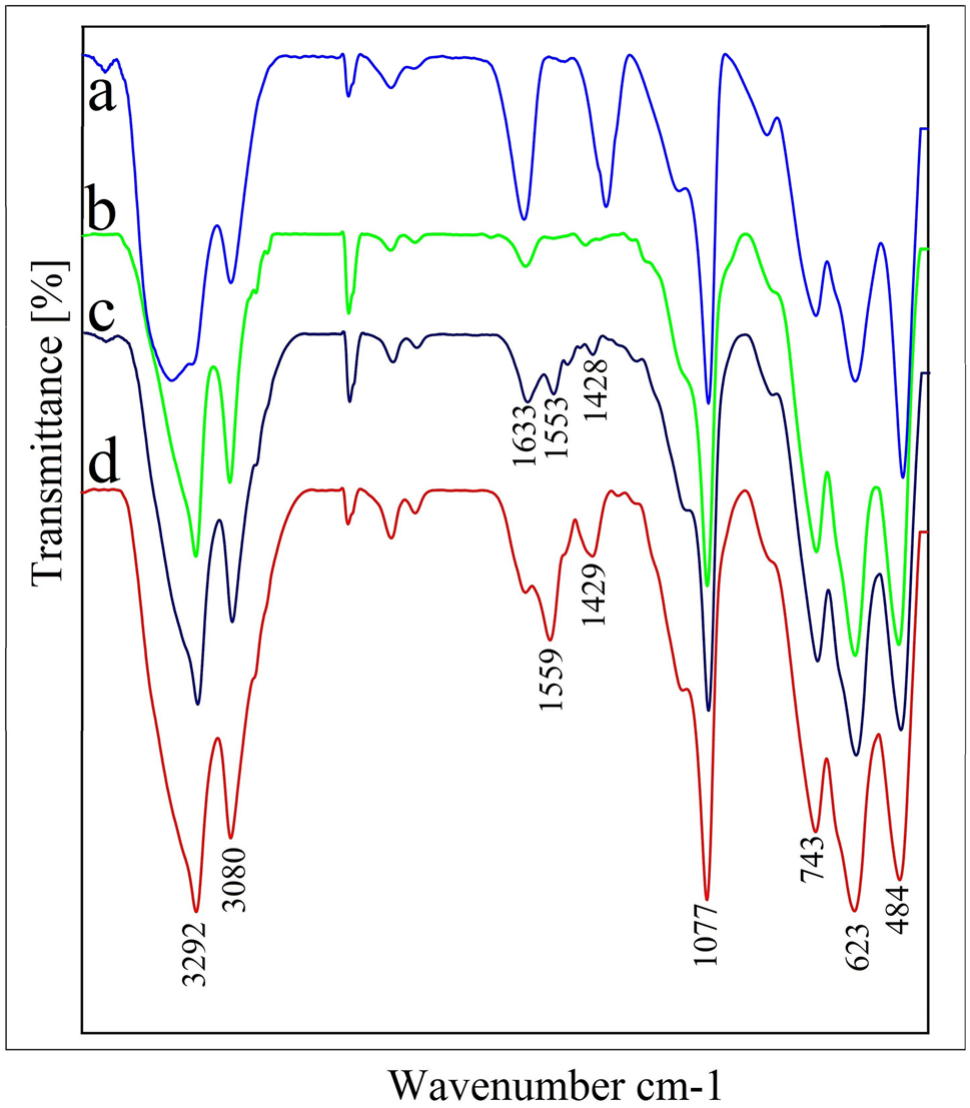
FT-IR spectra of (**a**) boehmite, (**b**) boehmite@CPTMS, (**c**) bis(PYT)@boehmite and (**d**) Sm-bis(PYT)@boehmite.

The FT-IR spectra of boehmite, boehmite@CPTMS, bis(PYT)@boehmite and Sm-bis(PYT)@boehmite are shown in Fig. 10. Three peaks at 484, 623, and 743 cm^−1^ can be related to the Al–O vibrations in the structure of boehmite^89^. The stretching vibration of Si–O–Si at 1077 cm^−1^^47,90^ indicates successful surface modification of boehmite nanoparticles. Also, the stretching vibration of C–H bonds can be observed at around 3000 cm^−1^ in the FT-IR spectra^90,91^—which not observed in the FT-IR spectrum of boehmite nanoparticles—indicating successful surface modification of boehmite nanoparticles. Moreover, two bands at 1559 cm^−1^ and 1429 cm^−1^ correspond to the aromatic C=C bonds^73,47^. The stretching vibration of C=N is observed at 1633 cm^−1^^73^.

### Catalytic study of Sm-bis(PYT)@boehmite

**Figure 11:**
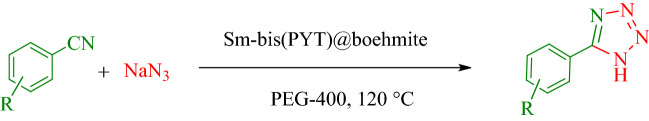
The general method used to synthesize 5-substituted 1H-tetrazoles in presence of Sm-bis(PYT)@boehmite nanocatalyst.

The catalytic application of Sm-bis(PYT)@boehmite is illustrated in the synthesis of tetrazole derivatives (Fig. 11). The best reaction conditions were found in the [3 + 2] cycloaddition of sodium azide (NaN_3_) and benzonitrile as the model reaction (Table 3). The model reaction cannot take place in the absence of Sm-bis(PYT)@boehmite catalyst (Table 3, entry 1). In this sense, the existence of Sm-bis(PYT)@boehmite is required to synthesize 5-substituted 1H-tetrazole derivatives. As accepted, the reaction proceeds in presence of the catalyst and, then, proceeds faster while increasing the amount of the catalyst. As shown, by increasing the amount of the catalyst up to 50 mg (containing 1.93 mol% of samarium), the model reaction can be completed within an acceptable time (Table 3, entry 3). As shown, when the amount of Sm-bis(PYT)@boehmite increased from 40 to 50 g, the turnover number (TOF) value improved more than twice. Among the several tested solvents (such as PEG-400, dimethyl sulfoxide (DMSO) and H_2_O), PEG-400 provided the best results in terms of TOF, reaction time, and isolated yield of the product (Table 3, entry 3). Furthermore, the effects of temperature and NaN_3_/benzonitrile ratio were checked on the model reaction and, accordingly, the best results were obtained at 120 °C with 1.4 mmol of NaN_3_ per 1 mmol of benzonitrile. As shown in Table 3, the best results (including TOF number, turnover number (TON), reaction time, and yield of the product) were obtained in presence of 50 mg of the catalyst in PEG-400 at 120 °C.

**Table 3:** Definition of the best reaction conditions for synthesizing 5-substituted 1H-tetrazoles in presence of Sm-bis(PYT)@boehmite.

Entry	Amount of the catalyst	Solvent	NaN_3_ (mmol)	Time (h)	Temperature (°C)	Yield (%)^a^	TON	TOF (/h)
1	–	PEG	1.4	2.5	120	N.R.^b^	–	–
2	40 mg, 1.54 mol%	PEG	1.4	4	120	87^c^	56.4	14.12
3	50, mg, 1.93 mol%	PEG	1.4	1.5	120	96	49.7	33.16
4	50, mg, 1.93 mol%	PEG	1.3	2	120	83	43.0	21.5
5	50, mg, 1.93 mol%	DMSO	1.4	1.7	120	68^c^	35.2	21.1
6	50, mg, 1.93 mol%	H_2_O	1.4	1.7	Reflux	Trace	–	–
7	50, mg, 1.93 mol%	PEG	1.4	1.7	100	41	21.2	12.7

^a^Isolated yield within 120 min.

^b^No reaction.

^c^Isolated by thin-layer chromatography.

To show the effect of the catalytic activity of the supported samarium, the [3 + 2] cycloaddition of benzonitrile with NaN_3_ under optimized conditions was studied in the presence of bare boehmite nanoparticles, boehmite@CPTMS, bis(PYT)@boehmite, and samarium(III) acetate salt. The obtained results were compared with the same reaction in the presence of Sm-bis(PYT)@boehmite (Table 4). As shown in Table 4, appropriate results can not be obtained for the synthesis of 5-phenyl-1H-tetrazole in the presence of unfunctionalized boehmite nanoparticles, boehmite@CPTMS, and bis(PYT)@boehmite, while 5-phenyl-1H-tetrazole was synthesized in the presence of Sm-bis(PYT)@boehmite in 90 min with a yield of 96%.

**Table 4:** Synthesis of 5-phenyl-1H-tetrazole in the presence of boehmite nanoparticles, boehmite@CPTMS, bis(PYT)@boehmite, samarium(III) acetate salt and Sm-bis(PYT)@boehmite.

Entry	Catalyst	Yield (%)^a^
1	Unfunctionalized boehmite nanoparticles	48
2	Boehmite@CPTMS	34
3	Bis(PYT)@boehmite	31
4	Samarium(III) acetate salt	90
5	Sm-bis(PYT)@boehmite	96

^a^Reaction conditions: benzonitrile (1 mmol), sodium azide (1.4 mmol) and catalyst (50 mg or 1.93 mol%) in PEG solvent at 120 °C for 1.5 h.

The scope of catalytic application of Sm-bis(PYT)@boehmite catalyst was extended in the [3 + 2] cycloaddition of NaN_3_ and other benzonitrile derivatives (Table 5). In this regard, benzonitriles having electron-withdrawing or electron-donating groups (on *para- meta- or ortho*-position of aromatic ring) were investigated, and all corresponding tetrazoles were synthesized in good yields.

**Table 5:** Synthesis of 5-substituted 1H-tetrazole derivatives catalyzed by Sm-bis(PYT)@boehmite.

Entry	Nitrile	Product	Time (h)	Yield (%)^a^	TON	TOF (h^-1^)	Melting point (°C)	Reported melting point (°C)
1			1.5	96	49.7	33.2	212–215	212–214^61^
2			3.83	94	48.7	12.7	180–183	180–183^60^
3			2	91	47.1	23.6	223–225	224–226^60^
4			1.5	91	47.1	31.4	211–212	209–212^70^
5			0.92	92	47.7	52.0	230–233	231–234^61^
6			2.33	96	49.7	21.3	260–263	261–264^61^
7			8.42	89	46.1	5.48	217–219	217–219^92^
8			18.5	93	48.2	2.60	260–262	259–261^48^
9			7.5	94	48.7	6.49	150–153	149–152^70^
10			4	95	49.2	12.3	254–256	253–257^93^
11			11.17	92	47.7	4.27	238–241	241–242^94^
12			16	93	48.2	3.01	247–250	248–251^93^
13			44	73	37.9	0.86	246–249	245–248^60^

^a^Isolated yield.

TON and TOF values are two valuable factors to evaluate the efficiency and practicality of catalysts. As shown in Table 5, all tetrazole products can be obtained with good TON and TOF numbers in the presence of Sm-bis(PYT)@boehmite catalyst. Therefore, one of the most important innovations of this work is the good TON and TOF values of the obtained products in the presence of Sm-bis(PYT)@boehmite catalyst.

**Figure 12:**
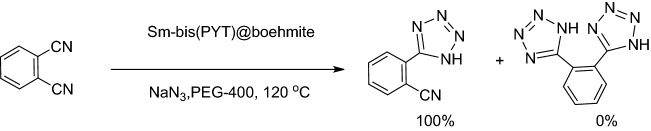
Homoselectivity of Sm-bis(PYT)@boehmite in the synthesis of 5-substituted 1H-tetrazoles from [3 + 2] cycloaddition of NaN_3_ with dicyano substituted derivatives.

As shown in Fig. 12, Sm-bis(PYT)@boehmite catalyst shows a good homoselectivity in the synthesis of tetrazoles through [3 + 2] cycloaddition of sodium azide and benzonitrile derivatives, which has two similar cyano groups in their structure such as phthalonitrile (Table 5, entry 4). As shown in Table 5 (entry 4) and Fig. 12, this strategy provided only monoaddition, which indicates an excellent homoselectivity of this catalyst.

**Figure 13:**
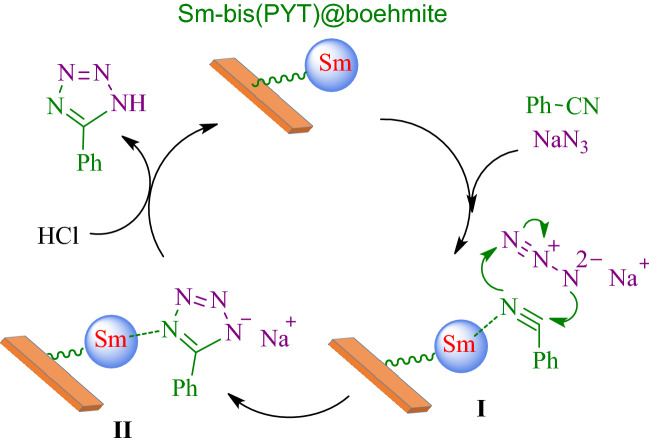
An expected mechanism for synthesizing 5-substituted 1H-tetrazoles in the presence of Sm-bis(PYT)@boehmite.

Based on authentically reported strategies for the synthesis of tetrazoles that are catalyzed by transition metal catalysts^19,95^, a catalytic cycle mechanism for the production of tetrazoles in the presence of Sm-bis(PYT)@boehmite catalyst is offered in Fig. 13.

### Comparison of the catalyst

The advantages of Sm-bis(PYT)@boehmite catalyst over previous catalysts was investigated in the synthesis of 5-phenyl-1H-tetrazole through [3 + 2] cycloaddition of benzonitrile with NaN_3_ in the presence of Sm-bis(PYT)@boehmite and previous catalysts (Table 6). As shown, Sm-bis(PYT)@boehmite catalyst affords 96% of the product in 90 min, which is better than the previous catalysts in terms of time and yields. Moreover, some of previous catalysts have several limitations or drawbacks, such as long reaction times, non or difficult separation of the catalysts and utilizing hazard solvents. Significantly, herein the synthesis of tetrazoles was introduced in a green solvent such as PEG, having a short reaction time and acceptable yield in presence of reusable Sm-bis(PYT)@boehmite catalyst.

**Table 6:** Comparison results of Sm-bis(PYT)@boehmite with other catalysts for the synthesis of 5-phenyl-1*H*-tetrazole.

Entry	Catalyst	Reaction conditions	Time (h)	Yield (%)	Refs.
1	CoY Zeolite	DMF, 120 °C	14	90	^96^
2	MCM-41@Cu	H_2_O, 80 °C	0.75	68	^97^
3	Cu–Zn alloy nanopowder	DMF, 135 °C	10	95	^94^
4	B(C_6_F_5_)_3_	DMF, 120 °C	8	94	^98^
5	Fe_3_O_4_@SiO_2_/Salen Cu(II)	DMF, 120 °C	7	90	^99^
6	Fe_3_O_4_/ZnS HNSs	DMF, 120 °C	24	81.1	^100^
7	Mesoporous ZnS	DMF, 120 °C	36	86	^101^
8	AgNO_3_	DMF, 120 °C	5	92	^102^
9	CuFe_2_O_4_	DMF, 120 °C	12	82	^103^
10	Nano ZnO/Co_3_O_4_	DMF, 130 °C	12	90	^104^
11	Cu-TBA@biochar	PEG-400, 130 °C	7	98	^73^
12	l-Cysteine-Pd@MCM-41	PEG-400, 100 °C	3	98	^78^
13	Ni-MP(AMP)_2_@Fe-biochar	PEG-400, 120 °C	4	92	^62^
14	Cu(II)-Adenine-MCM-41	PEG-400, 130 °C	5	92	^105^
15	Pd‐Arg@boehmite	PEG-400, 120 °C	7	97	^60^
16	ZrO-SB-APT@MCM-41	PEG-400, 120 °C	2	86	^106^
17	Cu-DABP@Fe_3_O_4_/MCM-41	PEG-400, 130 °C	2	99	^107^
18	Sm-bis(PYT)@boehmite	PEG-400, 120 °C	1.5	96	This work

### Recycling ability and leaching study of the catalyst

**Figure 14:**
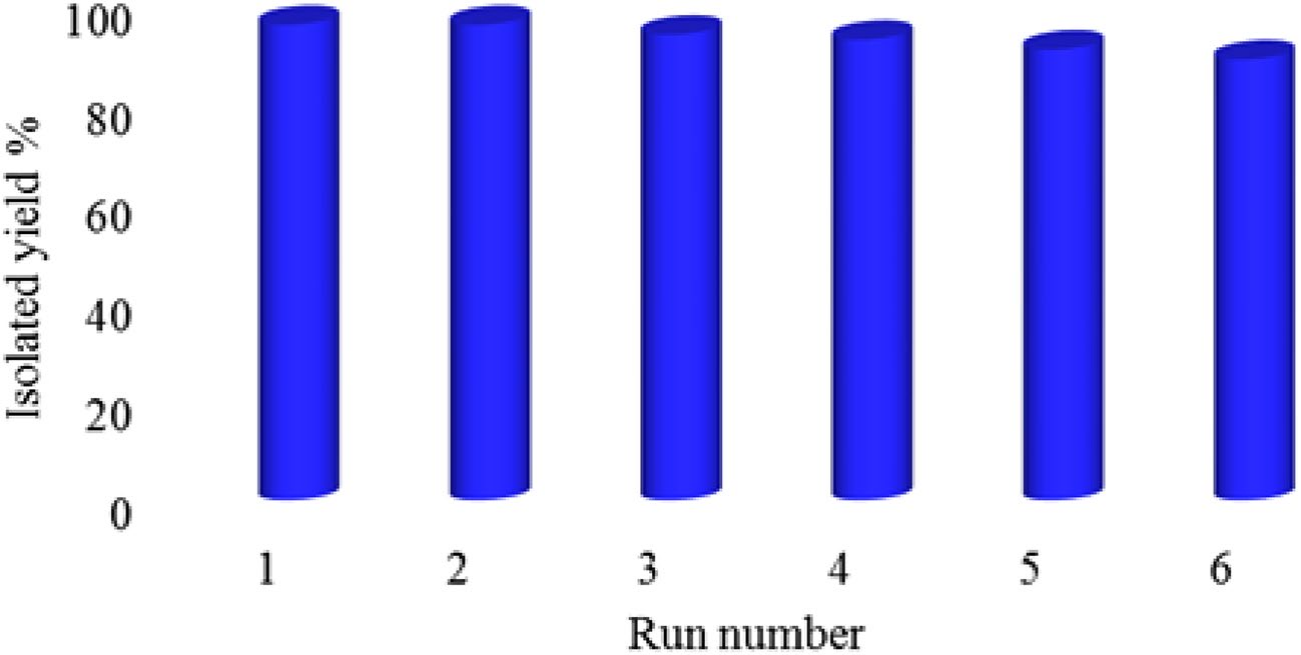
The recoverability and reusability of Sm-bis(PYT)@boehmite nanocatalyst in the synthesis of 5-phenyl-1H-tetrazole.

As mentioned, TGA analysis indicates that Sm-bis(PYT)@boehmite catalyst is stable at up to 300 °C. Therefore, the reusability of Sm-bis(PYT)@boehmite catalyst was investigated in the synthesis of 5-phenyl-1H-tetrazole through [3 + 2] cycloaddition of benzonitrile and NaN_3_. In this regard, at the end of the reaction, the reaction mixture was diluted and, then, the catalyst was recovered by filtration and reused in the next run. As shown in Fig. 14, Sm-bis(PYT)@boehmite catalyst can be recovered and reused for up to 6 runs without further activation.

The samarium leaching from Sm-bis(PYT)@boehmite catalyst in the synthesis of tetrazoles was studied applying hot filtration test and ICP-MS analysis. Thus, the [3 + 2] cycloaddition of benzonitrile and NaN_3_ in the presence of Sm-bis(PYT)@boehmite catalyst was repeated and, accordingly, the catalyst was removed after 0.75 h. The reaction mixture was allowed to continue without catalyst presence for up to 1.5 h. The reaction did not proceed after catalyst removing, signifying that samarium was not leached under reaction conditions.

In addition, this reaction was repeated and, at the end of the reaction, the catalyst was removed by simple filtration. Afterwards, the exact amount of the leached samarium in the reaction solution was calculated using ICP-MS analysis. No significant amount of the leached samarium was detected in the reaction solution.

The recovered Sm-bis(PYT)@boehmite nanocatalyst was characterized applying FT-IR, XRD, SEM, EDS, WDX and ICP-MS techniques.

**Figure 15:**
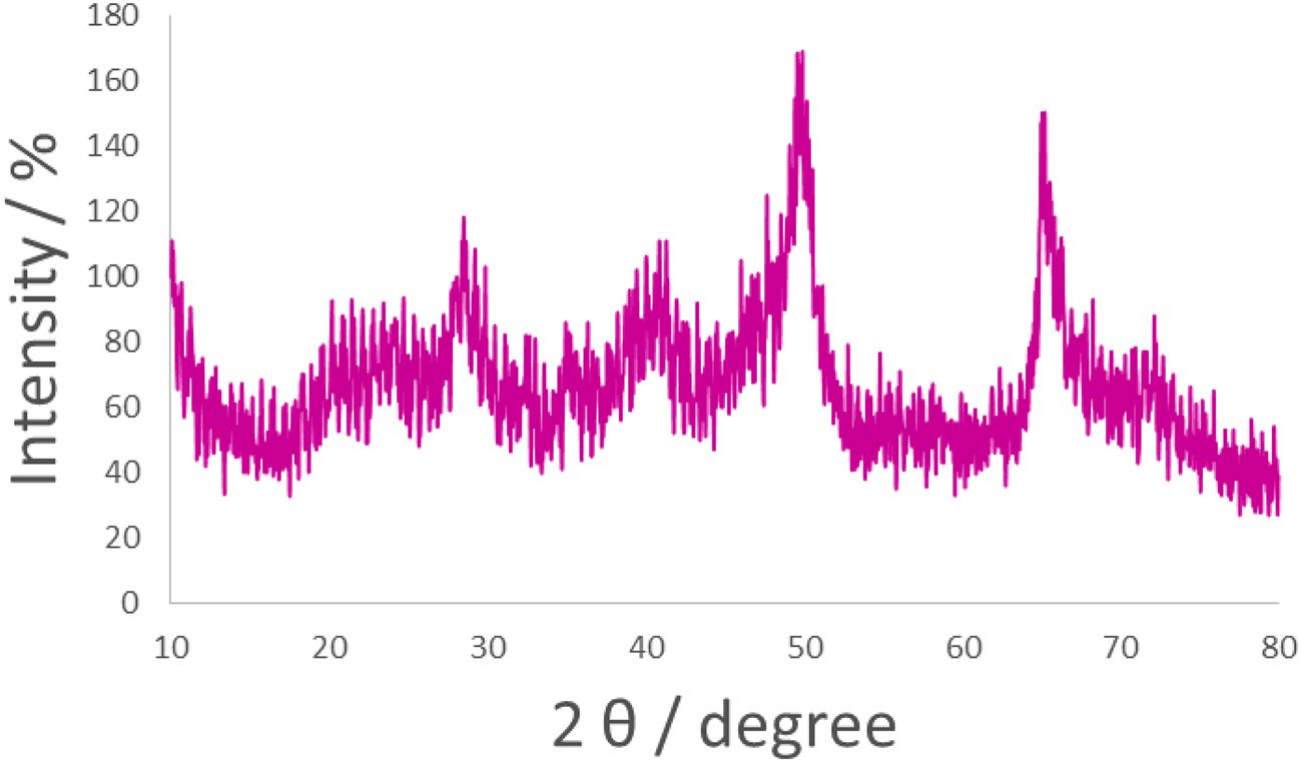
The powder XRD pattern of recovered Sm-bis(PYT)@boehmite.

The XRD pattern of the recovered Sm-bis(PYT)@boehmite nanocatalyst is illustrated in Fig. 15. A good agreement between fresh and recovered catalysts can be seen. No change was observed in the XRD pattern after reusing. These results verify the good stability of Sm-bis(PYT)@boehmite after reusing in the synthesis of tetrazoles.

**Figure 16:**
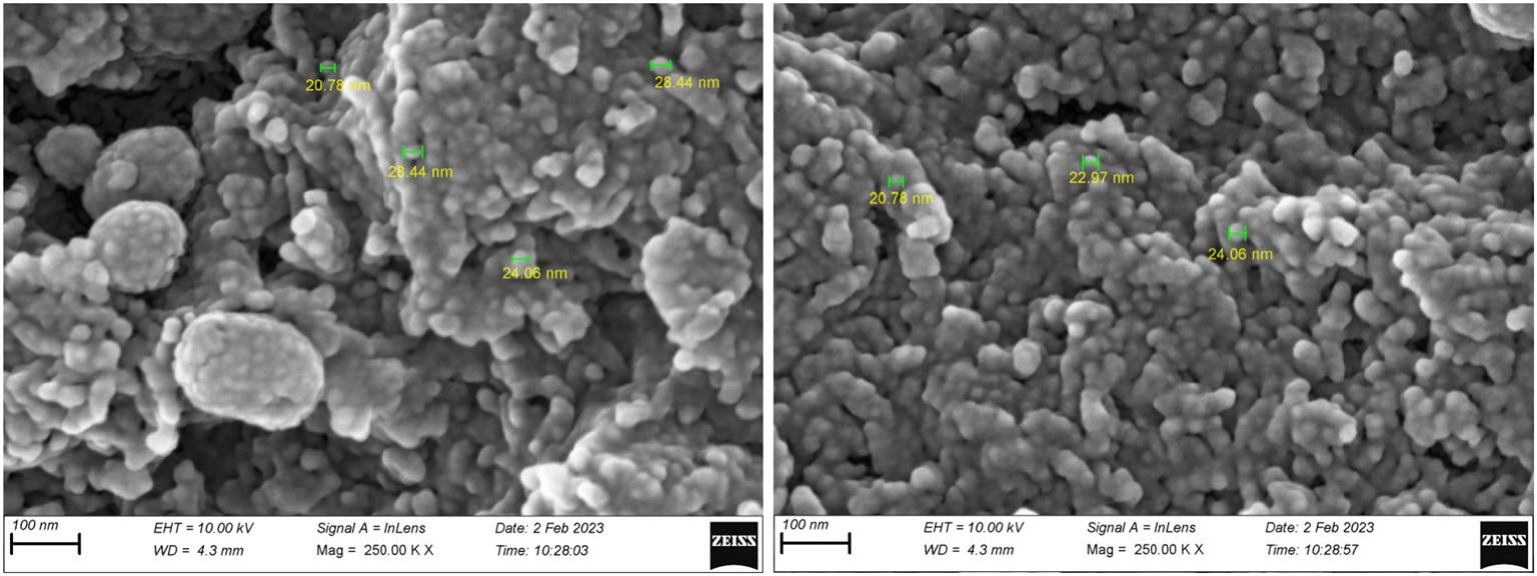
FESEM images of recovered Sm-bis(PYT)@boehmite.

The FE-SEM images of the recovered Sm-bis(PYT)@boehmite nanocatalyst are recorded using an electron microscope (FE-SEM ZEISS Sigma 300) model SIGMA VP from the Zeiss Company of German (Fig. 16). As shown, no significant change was observed in FESEM images of this catalyst after recovering in terms of particles size or shape. The SEM images indicate that the particles of the recovered Sm-bis(PYT)@boehmite have uniform spherical shapes and quite homogeneous diameter less than 70 nm, as compared to the catalyst before recovering.

**Figure 17:**
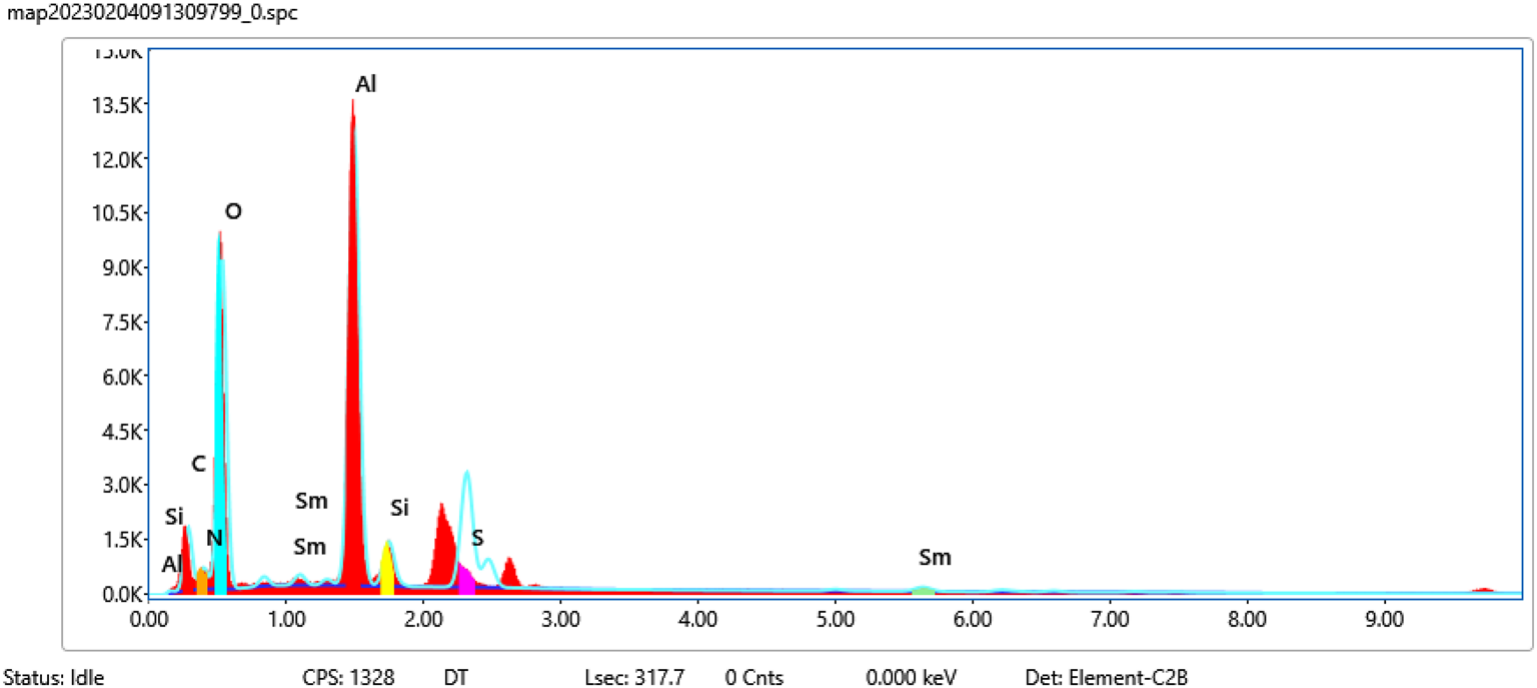
EDS diagram of recovered Sm-bis(PYT)@boehmite.

Furthermore, the electron microscope (FE-SEM ZEISS Sigma 300) model SIGMA VP from the Zeiss Company of German was employed for the elemental content of the recovered Sm-bis(PYT)@boehmite using EDS analysis. EDS diagram of the recovered Sm-bis(PYT)@boehmite is shown in Fig. 17. The EDS analysis indicated that the presence of all elements (e.g. Al, O, Si, C, N, S, and Sm elements) in the structure of the recovered Sm-bis(PYT)@boehmite shows a good agreement with the fresh catalyst.

**Figure 18:**
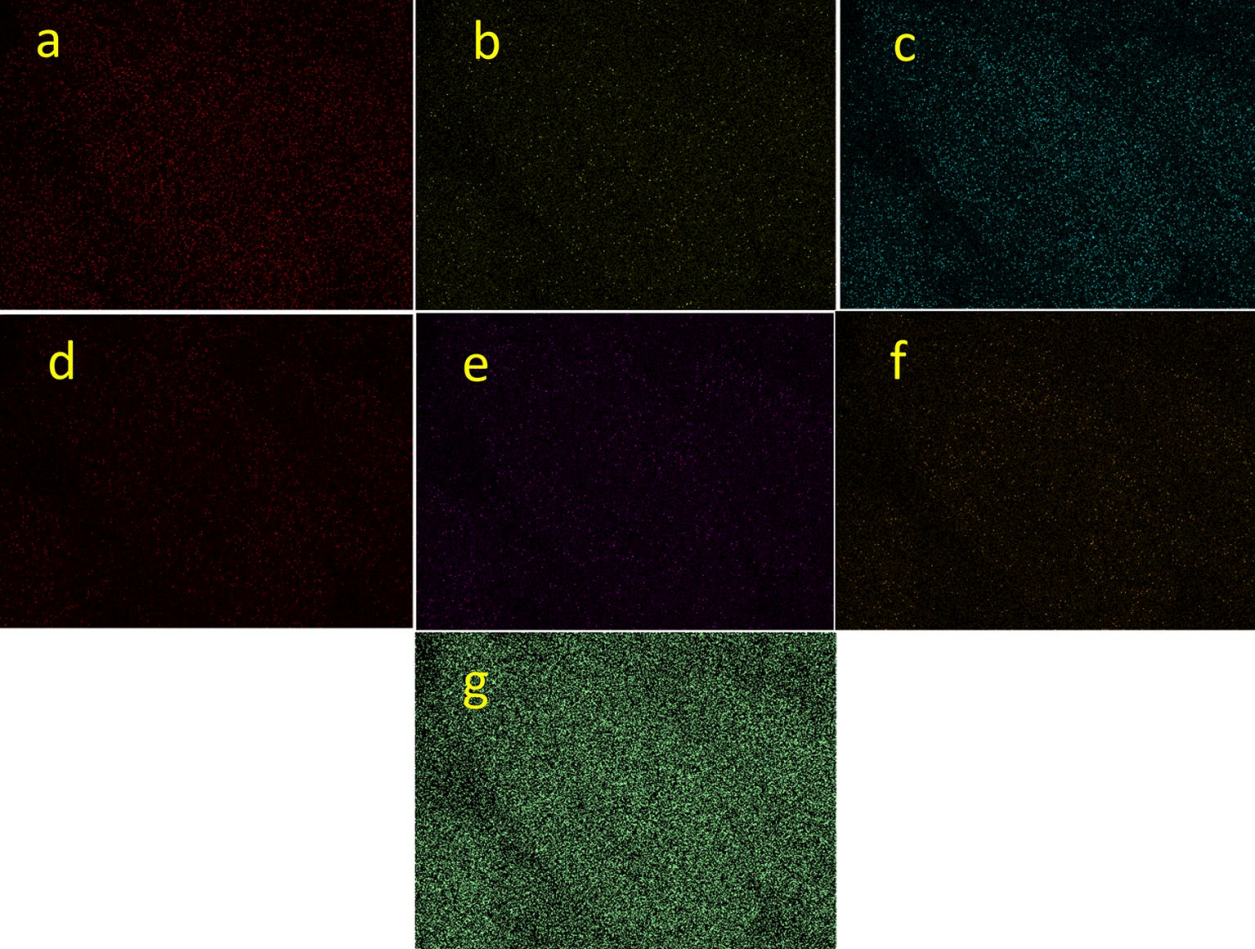
Elemental mapping of (**a**) Al, (**b**) Si, (**c**) O, (**d**) C, (**e**) S, (**f**) N and (**g**) Sm for recovered Sm-bis(PYT)@boehmite.

In addition, WDX analysis (Fig. 18) of the recovered Sm-bis(PYT)@boehmite illustrates a homogeneous distribution of Al, Si, O, C, N, S, and Sm in the structure of the recovered Sm-bis(PYT)@boehmite, as compared to this catalyst before recovering.

**Figure 19:**
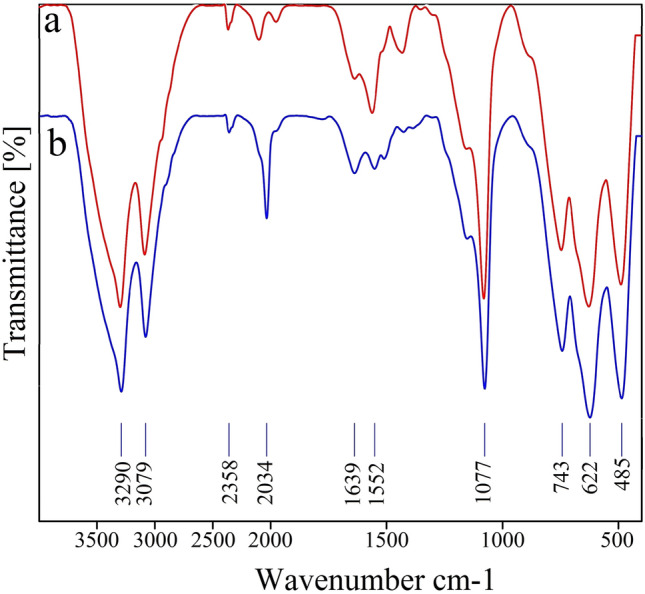
FT-IR spectra of (a) Sm-bis(PYT)@boehmite and (b) recovered Sm-bis(PYT)@boehmite.

Also, the FT-IR spectrum of the recovered Sm-bis(PYT)@boehmite was compared with the FT-IR spectrum of fresh Sm-bis(PYT)@boehmite. Excellent agreement was observed in the FT-IR spectra of the recovered and the fresh catalyst in terms of the position and shape of the stretching vibrations (Fig. 19). Therefore, Sm-bis(PYT)@boehmite is stable under reaction conditions during the synthesis of tetrazole derivatives.

FT-IR, XRD, SEM, EDS, WDX and ICP-MS analysis of the recovered Sm-bis(PYT)@boehmite proved the good stability of this catalyst after being reused in the synthesis of tetrazoles. Therefore, this catalyst is stable under reaction conditions of the tetrazoles synthesis. Therefore, it can be recovered and reused for several runs without any change in its structure.

## Methods

### Synthesis of 1,3-bis(pyridin-3-ylmethyl)thiourea ligand (3)

In a round-bottomed flask, 10 mmol of 3-(aminomethyl)pyridine (**1**) was added to 5 mmol of carbon disulfide (**2**) in water and, then, stirred at room temperature for 7 h. The reaction progress was consecutively monitored using thin-layer chromatography (TLC, ethyl acetate: n-hexane, 1:2). Since this reaction is exothermic, the temperature increases during the reaction and, therefore, the heat is sufficient to release H_2_S (**4**, confirmed by smell and lead acetate paper, which turns black). After completion of the reaction, the water-insoluble product (**3**) was filtered and, then, recrystallized from hot water and ethanol (1: 1 v/v).

### Synthesis of catalyst

In order to prepare boehmite, 50 mL of an aqueous solution of sodium hydroxide (6.490 g) was added to 30 mL of an aqueous solution of aluminum nitrate (20 g) as drop-by-drop under vigorous stirring. The resulting milky mixture was transferred to an ultrasonic bath for 3 h at room temperature. The resulting boehmite was filtered and, then, washed with distilled water. Subsequently, the obtained boehmite was kept in the oven at 220 °C for 4 h^60^.

The modified boehmite nanoparticles were synthesized according to a reported procedure in the literature^60^. In this regard, 1.5 g of boehmite nanoparticles was dispersed in n-hexane by sonication for up to 0.42 h and, then, 2 mL of (3-choloropropyl)triethoxysilane (CPTMS) was added to the reaction mixture. The obtained reaction mixture was stirred for 24 h under reflux conditions. The modified boehmite nanoparticles were produced through CPTMS (boehmite@CPTMS). The prepared boehmite@CPTMS was filtered, washed with ethanol, and dried at room temperature. In order to immobilize of 1,3-bis(pyridin-3-ylmethyl)thiourea ligand (**3**) on boehmite@CPTMS (bis(PYT)@boehmite), 1 g of boehmite@CPTMS was refluxed with **3** in toluene for 40 h. Afterwards, the obtained bis(PYT)@boehmite was isolated via simple filtration, washed with DMSO and ethanol and, then, dried at 60 °C. Finally, 1 g of bis(PYT)@boehmite was dispersed in EtOH, and then samarium(III) acetate was added to the mixture and stirred for 24 h under reflux conditions. The obtained catalyst (Sm-bis(PYT)@boehmite) was filtered, washed, and dried at 60 °C.

### General method for the synthesis of 5-substituted 1H-tetrazoles catalyzed by Sm-bis(PYT)@boehmite

[3 + 2] cycloaddition of sodium azide (NaN_3_) with organic nitrile compounds was selected for the synthesis of tetrazole derivatives in the presence of Sm-bis(PYT)@boehmite as a nanocatalyst. In this regard, 1.4 mmol of NaN_3_ and 1 mmol of nitrile were stirred in the presence of 50 mg of Sm-bis(PYT)@boehmite (1.93 mol% of samarium) in 2 mL of PEG-400 at 120 °C. At the end of the reaction, which was controlled using TLC, the reaction mixture was cooled down to room temperature. The reaction mixture was diluted by water and ethyl acetate and, then, Sm-bis(PYT)@boehmite nanocatalyst was separated by simple filtration. Afterwards, HCl (10 mL, 4 N) was added to the solution and, then, tetrazole products were extracted from ethyl acetate. The organic solvent was evaporated and dried using anhydrous sodium sulfate.

### Spectral data

**5-(2-Chlorophenyl)-1H-tetrazole** ^1^H NMR (400 MHz, DMSO): δ_H_ = 7.81–7.78 (d, *J* = 12 Hz, 1H), 7.72–7.69 (d, *J* = 12 Hz, 1H), 7.64–7.60 (t, *J* = 8 Hz, 1H), 7.57–7.52 (t, *J* = 8 Hz, 1H) ppm (Fig. S1, Supplementary Material).

**5-(4-Nitrophenyl)-1H-tetrazole** ^1^H NMR (400 MHz, DMSO): δ_H_ = 16.55 (br, 1H), 8.46–8.43 (d, *J* = 12 Hz, 2H), 8.31–8.28 (d, *J* = 12 Hz, 2H) ppm (Fig. S2, Supplementary Material).

## Conclusion

In Conclusion, natural and environmentally friendly boehmite nanoparticles were synthesized using sodium hydroxide and aluminum nitrate in an aqueous solution. The surface of boehmite nanoparticles was modified applying CPTMS. Afterwards, a new samarium complex of 1,3-bis(pyridin-3-ylmethyl)thiourea was stabilized on the modified boehmite nanoparticles (Sm-bis(PYT)@boehmite). Moreover, Sm-bis(PYT)@boehmite was used as an efficient, environmentally friendly, and reusable nanocatalyst in the homoselective synthesis of tetrazole derivatives from [2 + 3] cycloaddition reaction of NaN_3_ and nitriles in PEG-400 solvent. Significantly all tetrazoles were obtained with excellent yields and good TON and TOF values within short reaction times. This nanocatalyst was characterized using several techniques, such as FT-IR, SEM, EDS, WDX, TGA, XRD, DLS, ICP-MS, and BET. This nanocatalyst showed high activity, good selectivity, stability, and recyclability in the synthesis of tetrazole derivatives. Sm-bis(PYT)@boehmite was recovered and reused for six times without notable loss of catalytic efficiency. After being reused, the stability and heterogeneity of the catalyst were investigated using techniques such as FT-IR, XRD, SEM, EDS, WDX and ICP-MS analysis.

## Supplementary Information

Supplementary Information.

## Data Availability

Data available in article supplementary material; the data that support the findings of this study are available in the supplementary material of this article.

## References

[1] Sadjadi S, Abedian-Dehaghani N, Heravi M (2022). Pd on cyclotriphosphazen-hexa imine decorated boehmite as an efficient catalyst for hydrogenation of nitro arenes under mild reaction condition. Sci. Rep..

[2] Moradi P, Hajjami M (2022). Stabilization of ruthenium on biochar-nickel magnetic nanoparticles as a heterogeneous, practical, selective, and reusable nanocatalyst for the Suzuki C–C coupling reaction in water. RSC Adv..

[3] Balakrishnan M, Batra VS, Hargreaves JSJ, Pulford ID (2011). Waste materials—Catalytic opportunities: an overview of the application of large scale waste materials as resources for catalytic applications. Green Chem..

[4] Dhenadhayalan N, Lin KC, Saleh TA (2020). Recent advances in functionalized carbon dots toward the design of efficient materials for sensing and catalysis applications. Small.

[5] Yue Y (2017). Template-free synthesis and catalytic applications of microporous and hierarchical ZSM-5 zeolites from natural aluminosilicate minerals. Ind. Eng. Chem. Res..

[6] Sardarian AR, DindarlooInaloo I, Zangiabadi M (2018). Selective synthesis of secondary arylcarbamates via efficient and cost effective copper-catalyzed mono arylation of primary carbamates with aryl halides and arylboronic acids. Catal. Lett..

[7] Dindarloo Inaloo I, Esmaeilpour M, Majnooni S, Oveisi AR (2020). Nickel-catalyzed synthesis of N-(hetero)aryl carbamates from cyanate salts and phenols activated with cyanuric chloride. ChemCatChem.

[8] Dindarloo Inaloo I, Majnooni S, Eslahi H, Esmaeilpour M (2020). Efficient nickel(II) immobilized on EDTA-modified Fe_3_O_4_@SiO_2_ nanospheres as a novel nanocatalyst for amination of heteroaryl carbamates and sulfamates through the cleavage of C-O bond. Mol. Catal..

[9] Dindarloo Inaloo I, Majnooni S, Eslahi H, Esmaeilpour M (2020). Air-stable Fe_3_O_4_@SiO_2_-EDTA-Ni(0) as an efficient recyclable magnetic nanocatalyst for effective suzuki-miyaura and heck cross-coupling via aryl sulfamates and carbamates. Appl. Organomet. Chem..

[10] Kloprogge JT, Ruan HD, Frost RL (2002). Thermal decomposition of bauxite minerals: Infrared emission spectroscopy of gibbsite, boehmite and diaspora. J. Mater. Sci..

[11] Souza Santos P, Vieira Coelho AC, Souza Santos H, Kunihiko Kiyohara P (2009). Hydrothermal synthesis of well-crystallised boehmite crystals of various shapes. Mat. Res..

[12] Jabbari A, Moradi P, Hajjami M, Tahmasbi B (2022). Tetradentate copper complex supported on boehmite nanoparticles as an efficient and heterogeneous reusable nanocatalyst for the synthesis of diaryl ethers. Sci. Rep..

[13] Xie Y, Kocaefe D, Kocaefe Y, Cheng J, Liu W (2016). The effect of novel synthetic methods and parameters control on morphology of nano-alumina particles. Nanosc. Res. Lett..

[14] Peintinger MF, Kratz MJ, Bredow T (2014). Quantum-chemical study of stable, meta-stable and high-pressure alumina polymorphs and aluminum hydroxides. J. Mater. Chem. A.

[15] Rajabi L, Derakhshan AA (2010). Room temperature synthesis of boehmite and crystallization of nanoparticles: effect of concentration and ultrasound. Sci. Adv. Mater..

[16] Walmiki T, Subagjo S, Lismana KR, Fuadi K (2011). Synthesis of γ-Al_2_O_3_ catalyst support from kaolin of indonesian origin. ITB J. Eng. Sci..

[17] Bruhne S, Gottlieb S, Assmus W, Alig E, Schmidt MU (2008). Atomic structure analysis of nanocrystalline boehmite AlO(OH). Cryst. Growth Des..

[18] Karger-Kocsis J, Lendvai L (2018). Polymer/boehmite nanocomposites: A review. J. Appl. Polym. Sci..

[19] Tahmasbi B, Ghorbani-Choghamarani A, Moradi P (2020). Palladium fabricated on boehmite as an organic–inorganic hybrid nanocatalyst for C-C cross coupling and homoselective cycloaddition reactions. N. J. Chem..

[20] Fankhänel J, Sinapius M, Ziegmann G (2021). Mechanical properties of boehmite evaluated by atomic force microscopy experiments and molecular dynamic finite element simulations. Acting Principles of Nano-Scaled Matrix Additives for Composite Structures. Research Topics in Aerospace.

[21] Candela L, Perlmutter DD (1992). Kinetics of boehmite formation by thermal decomposition of gibbsite. Ind. Eng. Chem. Res..

[22] Mishra D, Anand S, Panda RK, Das RP (2000). Hydrothermal preparation and characterization of boehmites. Mater. Lett..

[23] Nguefack M, Popa AF, Rossignol S, Kappenstein C (2003). Preparation of alumina through a sol–gel process. Synthesis, characterization, thermal evolution and model of intermediate boehmite. Phys. Chem. Chem. Phys..

[24] Ghorbani-Choghamarani A, Moradi P, Tahmasbi B (2019). Modification of boehmite nanoparticles with Adenine for the immobilization of Cu(II) as organic–inorganic hybrid nanocatalyst in organic reactions. Polyhedron.

[25] Mohammadi M, Khodamorady M, Tahmasbi B, Bahrami K, Ghorbani-Choghamarani A (2021). Boehmite nanoparticles as versatile support for organic–inorganic hybrid materials: Synthesis, functionalization, and applications in eco-friendly catalysis. J. Ind. Eng. Chem..

[26] Ghorbani-Choghamarani A, Seydyosefi Z, Tahmasbi B (2018). Tribromide ion supported on boehmite nanoparticles as a reusable catalyst for organic reactions. C. R. Chimie.

[27] Baran NY, Baran T, Nasrollahzadeh M, Varma RS (2019). Pd nanoparticles stabilized on the Schiff base-modified boehmite: Catalytic role in Suzuki coupling reaction and reduction of nitroarenes. J. Organometal. Chem..

[28] Jani MA, Bahrami K (2020). Synthesis of 5-substituted 1H-tetrazoles and oxidation of sulfides by using boehmite nanoparticles/nickel-curcumin as a robust and extremely efficient green nanocatalyst. Appl Organomet Chem..

[29] Yan Z (2018). Enhanced room-temperature catalytic decomposition of formaldehyde on magnesium-aluminum hydrotalcite/boehmite supported platinum nanoparticles catalyst. J. Colloid Interface Sci..

[30] Mirzaee M, Bahramian B, Shahraki M, Moghadam H, Mirzaee A (2018). Molybdenum containing catalysts grafted on functionalized hydrous zirconia nano-particles for epoxidation of alkenes. Catal. Lett..

[31] Mirzaee M, Bahramian B, Gholampour P, Teymouri S, Khorsand T (2019). Preparation and characterization of Fe_3_O_4_@Boehmite core-shell nanoparticles to support molybdenum or vanadium complexes for catalytic epoxidation of alkenes. Appl. Organomet. Chem..

[32] Mohammadinezhad A, Akhlaghinia B (2017). Fe_3_O_4_@Boehmite-NH_2_-CoII NPs: an inexpensive and highly efficient heterogeneous magnetic nanocatalyst for the Suzuki-Miyaura and Heck-Mizoroki cross-coupling reactions. Green Chem..

[33] Ghorbani-Choghamarani A, Heidarnezhad Z, Tahmasbi B (2019). New complex of copper on boehmite nanoparticles as highly efficient and reusable nanocatalyst for synthesis of sulfides and ethers. ChemistrySelect.

[34] Huang G (2009). Immobilization of manganese tetraphenylporphyrin on boehmite and its catalysis for aerobic oxidation of cyclohexane. Appl. Catal. A Gen..

[35] Huang G (2011). Catalysis behavior of boehmite-supported iron tetraphenylporphyrins with nitro and methoxyl substituents for the aerobic oxidation of cyclohexane. J. Mol. Catal. A Chem..

[36] Park IS (2005). Rhodium nanoparticles entrapped in boehmite nanofibers: Recyclable catalyst for arenehydrogenation under mild conditions. Chem. Commun..

[37] Zhou YF (2009). Efficient hydrogenation of methyl propionate over boehmite-supported Ru–Pt catalyst. Chem. Lett..

[38] Claude V, Mahy JG, Lohay T, Geens J, Lambert SD (2022). Coating process of honeycomb cordierite support with Ni/boehmite gels. Processes.

[39] Panda AP, Jha U, Swain SK (2020). Synthesis of nanostructured copper oxide loaded boehmite (CuO_Boehmite) for adsorptive removal of As(III/V) from aqueous solution. J. Water Process. Eng..

[40] Li X (2022). Influence of melamine cyanurate and boehmite on flame retardancy of PA6. Iran. Polym. J..

[41] Wang H, Lundin STB, Takanabe K, Oyama ST (2022). Synthesis of size-controlled boehmite sols: Application in high-performance hydrogen-selective ceramic membranes. Mater. Chem. A.

[42] Nettar CB, Bhowmik RN, Sinha AK (2022). A comparative study of the lattice structure, optical band gap, electrical conductivity and polarization at different stages of the heat treatment of chemical routed Al(OH)_3_. Ceram. Int..

[43] Liang Z (2022). Mechanistic understanding of the aspect ratio-dependent adjuvanticity of engineered aluminum oxyhydroxide nanorods in prophylactic vaccines. Nano Today.

[44] Szczęśniak B, Choma J, Jaroniec M (2021). Facile mechanochemical synthesis of highly mesoporous γ-Al_2_O_3_ using boehmite. Microporous Mesoporous Mater..

[45] Ghadermazi M, Molaei S (2022). Synthesis of SBA-15@3,4,5-tri hydroxyphenyl acetic@ Tb for the facile synthesis of 5-substituted 1 H-tetrazoles. Catal. Surv. Asia..

[46] Molaei S, Ghadermazi M (2022). Introduction of Ni into mesoporous MCM-41: A new recyclable catalyst for the synthesis of 5-substituted 1H-tetrazoles and the selective oxidation of sulfides. J. Porous Mater..

[47] Nikoorazm M, Noori N, Tahmasbi B, Faryadi S (2017). A palladium complex immobilized onto mesoporous silica: A highly efficient and reusable catalytic system for carbon–carbon bond formation and anilines synthesis. Transit. Met. Chem..

[48] Nikoorazm M, Tahmasbi B, Gholami S, Moradi P (2020). Copper and nickel immobilized on cytosine@MCM-41: As highly efficient, reusable and organic–inorganic hybrid nanocatalysts for the homoselective synthesis of tetrazoles and pyranopyrazoles. Appl. Organomet. Chem..

[49] Koolivand M, Nikoorazm M, Ghorbani-Choghamarani A, Tahmasbi B (2021). Cu–citric acid metal–organic framework: Synthesis, characterization and catalytic application in Suzuki-Miyaura cross-coupling reaction and oxidation of sulfides. Appl. Organomet. Chem..

[50] Ghorbani-Choghamarani A, Hajjami M, Tahmasbi B, Noori N (2016). Boehmite silica sulfuric acid: As a new acidic material and reusable heterogeneous nanocatalyst for the various organic oxidation reactions. J. Iran. Chem. Soc..

[51] Ghorbani-Choghamarani A, Moradi P, Tahmasbi B (2019). Nickel(II) immobilized on dithizone–boehmite nanoparticles: As a highly eicient and recyclable nanocatalyst for the synthesis of polyhydroquinolines and sulfoxidation reaction. J. Iran. Chem. Soc..

[52] Corcho-Valdés AL (2022). Carbon nanotubes in organic catalysis. Carbon Comp. Catal..

[53] Ghorbani-Choghamarani A, Azadi G, Tahmasbi B, Hadizadeh-Hafshejani M, Abdi Z (2014). Practical and versatile oxidation of sulfides into sulfoxides and oxidative coupling of thiols using polyvinylpolypyrrolidonium tribromide. Phosphorus Sulfur Silicon Relat. Elem..

[54] Moeini N, Ghadermazi M, Molaei S (2022). Synthesis and characterization of magnetic Fe_3_O_4_@Creatinine@Zr nanoparticles as novel catalyst for the synthesis of 5-substituted 1H-tetrazoles in water and the selective oxidation of sulfides with classical and ultrasonic methods. J. Mol. Struct..

[55] Moeini N, Molaei S, Ghadermazi M (2022). Selective oxidation of sulfides and synthesis of 5-substituted 1H-tetrazoles on Ce (III) immobilized CoFe_2_O_4_ as a magnetically separable, highly active, and reusable nanocatalyst. Res. Chem. Intermed..

[56] Shiri L, Tahmasbi B (2017). Tribromide ion immobilized on magnetic nanoparticles as an efficient catalyst for the rapid and chemoselective oxidation of sulfides to sulfoxides. Phosphorus Sulfur Silicon Relat. Elem..

[57] Ghorbani-Choghamarani A, Tahmasbi B, Noori N, Faryadi S (2017). Pd–S-methylisothiourea supported on magnetic nanoparticles as an efficient and reusable nanocatalyst for Heck and Suzuki reactions. C. R. Chimie.

[58] Molaei S, Moeini N, Ghadermazi M (2022). Synthesis of CoFe_2_O_4_ @Amino glycol/Gd nanocomposite as a high-efficiency and reusable nanocatalyst for green oxidation of sulfides and synthesis of 5-substituted 1H-tetrazoles. J. Organomet. Chem..

[59] Zarchi MAK, Nazem F (2012). Using a polymer-supported azide ion in [2+ 3] cycloaddition reaction of azide ion with nitriles. J. Appl. Polym. Sci..

[60] Tahmasbi B, Ghorbani-Choghamarani A (2017). First report of the direct supporting of palladium–arginine complex on boehmite nanoparticles and application in the synthesis of 5-substituted tetrazoles. Appl. Organomet. Chem..

[61] Jabbari A, Tahmasbi B, Nikoorazm M, Ghorbani-Choghamarani A (2018). A new Pd-Schiff-base complex on boehmite nanoparticles: Its application in Suzuki reaction and synthesis of tetrazoles. Appl. Organometal. Chem..

[62] Moradi P, Hajjami M (2021). Magnetization of biochar nanoparticles as a novel support for fabrication of organo nickel as a selective, reusable and magnetic nanocatalyst in organic reactions. New J. Chem..

[63] Ghodsinia SSE, Akhlaghinia B (2015). A rapid metal free synthesis of 5-substituted-1H-tetrazoles using cuttlebone as a natural high effective and low cost heterogeneous catalyst. RSC Adv..

[64] Demko ZP, Sharpless KB (2001). Preparation of 5-substituted 1H-tetrazoles from nitriles in water. J. Org. Chem..

[65] Ghadermazi M, Molaei S, Ghadermazi N (2021). Introduction of Fe into mesoporous MCM-41 for the synthesis of 5-substituted 1H-Tetrazoles from aryl nitriles in water. Microporous Mesoporous Mater..

[66] Molaei S, Ghadermazi M, Moeini N (2022). Selectivity adjustment of Fe_3_O_4_ MNPs based silver catalyst in oxidation of sulfides with classical and ultrasonic methods and synthesis of 5-substituted 1H-tetrazoles from aryl nitriles in water. Appl. Surf. Sci..

[67] Ghadermazi M, Molaei S (2023). Synthesis of Sm (III) complex immobilized in MCM-41: A new heterogeneous catalyst for the facile synthesis of 5-substituted 1H-tetrazoles via [3 + 2] cycloaddition of nitriles and sodium azide. Inorg. Chem. Commun..

[68] Molaei S, Ghadermazi M (2021). Cu attached functionalized mesoporous MCM-41: a novel heterogeneous nanocatalyst for eco-friendly one-step thioether formation reaction and synthesis of 5-substituted 1H-tetrazoles. Res. Chem. Intermed..

[69] Moradi P (2022). Investigation of Fe_3_O_4_@boehmite NPs as efficient and magnetically recoverable nanocatalyst in the homoselective synthesis of tetrazoles. RSC Adv..

[70] Tahmasbi B, Nikoorazm M, Moradi P, Abbasi Tyula Y (2022). A Schiff base complex of lanthanum on modified MCM-41 as a reusable nanocatalyst in the homoselective synthesis of 5-substituted 1H-tetrazoles. RSC Adv..

[71] Neochoritis CG, Zhao T, Dömling A (2019). Tetrazoles via multicomponent reactions. Chem. Rev..

[72] Hamrahian SA, Salehzadeh S, Rakhtshah J, Haji Babaei F, Karami N (2019). Preparation, characterization and catalytic application of molybdenum Schiff-base complex immobilized on silica-coated Fe_3_O_4_ as a reusable catalyst for the synthesis of pyranopyrazole derivatives. Appl. Organomet. Chem..

[73] Moradi P, Hajjami M, Tahmasbi B (2020). Fabricated copper catalyst on biochar nanoparticles for the synthesis of tetrazoles as antimicrobial agents. Polyhedron.

[74] Rezaei F, Amrollahi MA, Khalifeh R (2019). Design and synthesis of Fe_3_O_4_@SiO_2_/aza-crown ether-Cu(II) as a novel and highly efficient magnetic nanocomposite catalyst for the synthesis of 1,2,3-triazoles, 1-substituted 1H-tetrazoles and 5-substituted 1H-tetrazoles in green solvents. Inorganica Chim. Acta..

[75] Samanta PK (2019). Mesoporous silica supported samarium as recyclable heterogeneous catalyst for synthesis of 5-substituted tetrazole and 2-substituted benzothiazole. J. Porous Mater..

[76] Akbarzadeh P, Koukabi N, Kolvari E (2019). Three-component solvent-free synthesis of 5-substituted-1H-tetrazoles catalyzed by unmodified nanomagnetite with microwave irradiation or conventional heating. Res. Chem. Intermed..

[77] Maleki A, Niksefat M, Rahimi J, Azadegan S (2019). Facile synthesis of tetrazolo [1, 5-a] pyrimidine with the aid of an effective gallic acid nanomagnetic catalyst. Polyhedron.

[78] Nikoorazm M, Moradi P, Noori N (2020). L-cysteine complex of palladium onto mesoporous channels of MCM-41 as reusable, homoselective and organic–inorganic hybrid nanocatalyst for the synthesis of tetrazoles. J. Porous Mater..

[79] Ghorbani-Choghamarani A, Tahmasbi B, Noori N, Ghafouri-nejad R (2017). A new palladium complex supported on magnetic nanoparticles and applied as an catalyst in amination of aryl halides, Heck and Suzuki reactions. J. Iran. Chem. Soc..

[80] Nikoorazm M, Moradi P, Noori N, Azadi G (2021). L-Arginine complex of copper on modified core–shell magnetic nanoparticles as reusable and organic–inorganic hybrid nanocatalyst for the chemoselective oxidation of organosulfur compounds. J. Iran. Chem. Soc..

[81] Rezaei A, Ghorbani-Choghamarani A, Tahmasbi B (2022). Synthesis and characterization of nickel metal-organic framework including 4,6-diamino-2-mercaptopyrimidine and its catalytic application in organic reactions. Catal. Lett..

[82] Chen Y (2018). Epoxy/α-alumina nanocomposite with decreased dielectric constant and dielectric loss. Polym. Compos..

[83] Chen Y (2017). Epoxy/α-alumina nanocomposite with high electrical insulation performance. Prog. Nat. Sci. Mater. Int..

[84] Ortega-Franqueza M, Ivanova S, Isabel Domínguez M, Ángel Centeno M (2021). Mesoporous carbon production by nanocasting technique using boehmite as a template. Catalysts.

[85] Raso R, García L, Ruiz J, Oliva M, Arauzo J (2020). Study of Ni/Al-Fe catalyst stability in the aqueous phase hydrogenolysis of glycerol. Catalysts.

[86] Santos RP (2012). Investigation of the nature of V-species on alumina modified by alkali cations: Development of multi-functional DeSO_x_ catalysts. Appl. Catal. A: Gen..

[87] Bhusan Mishra B, Devi N, Sarangi K (2020). Recovery of samarium and cobalt from Sm–Co magnet waste using a phosphonium ionic liquid cyphos IL 104. J. Sustain. Metall..

[88] Moradi P, Hajjami M (2021). Magnetization of graphene oxide nanosheets using nickel magnetic nanoparticles as a novel support for the fabrication of copper as a practical, selective, and reusable nanocatalyst in C–C and C–O coupling reactions. RSC Adv..

[89] Ghorbani-Choghamarani A, Seydyosefi Z, Tahmasbi B (2018). Zirconium oxide complex anchored on boehmite nanoparticles as highly reusable organometallic catalyst for C-S and C–O coupling reactions. Appl. Organometal. Chem..

[90] Moradi P, Zarei B, Abbasi Tyula Y, Nikoorazm M (2023). Novel neodymium complex on MCM-41 magnetic nanocomposite as a practical, selective and returnable nanocatalyst in the synthesis of tetrazoles with antifungal properties in agricultural. Appl. Organomet. Chem..

[91] Jabbari A, Nikoorazm M, Moradi P (2023). A V(O)-Schiff-base complex on MCM-41 as an efficient, reusable, and chemoselective nanocatalyst for the oxidative coupling of thiols and oxidation of sulfides. Res. Chem. Intermed..

[92] Aqeel Ashraf M, Liu Z, Li C, Zhang D (2020). Fe_3_O_4_@L-lysine-Pd(0) organic–inorganic hybrid: As a novel heterogeneous magnetic nanocatalyst for chemo and homoselective [2 + 3] cycloaddition synthesis of 5-substituted 1H-tetrazoles. Appl. Organomet. Chem..

[93] Aali E, Gholizadeh M, Noroozi-Shad N (2022). 1-Disulfo-[2,2-bipyridine]-1,1-diium chloride ionic liquid as an efficient catalyst for the green synthesis of 5-substituted 1H-tetrazoles. J. Mol. Struct..

[94] Aridoss G, Laali KK (2011). Highly efficient synthesis of 5-substituted 1H-tetrazoles catalyzed by Cu–Zn alloy nanopowder, conversion into 1,5- and 2,5-disubstituted tetrazoles, and synthesis and NMR studies of new tetrazolium ionic liquids. Eur. J. Org. Chem..

[95] Abrishami F, Ebrahimikia M, Rafiee F (2015). Synthesis of 5-substituted 1H-tetrazoles using a recyclable heterogeneous nanonickel ferrite catalyst. Appl. Organomet. Chem..

[96] Rama V, Kanagaraj K, Pitchumani K (2011). Syntheses of 5-substituted 1H-tetrazoles catalyzed by reusable CoY zeolite. J. Org. Chem..

[97] Ghadermazi M, Molaei S, Khorami S (2022). Synthesis, characterization and catalytic activity of copper deposited on MCM-41 in the synthesis of 5-substituted 1H-tetrazoles. J. Porous Mater..

[98] Kumar Prajapti S, Nagarsenkar A, Nagendra Babu B (2014). An efficient synthesis of 5-substituted 1H-tetrazoles via B(C_6_F_5_)_3_ catalyzed [3+2] cycloaddition of nitriles and sodium azide. Tetrahedron Lett..

[99] Dehghani F, Sardarian AR, Esmaeilpour M (2013). Salen complex of Cu(II) supported on superparamagnetic Fe_3_O_4_@SiO_2_ nanoparticles: An efficient and recyclable catalyst for synthesis of 1- and 5-substituted 1H-tetrazoles. J. Organomet. Chem..

[100] Qi G, Liu W, Bei Z (2011). Fe_3_O_4_/ZnS hollow nanospheres: A highly efficient magnetic heterogeneous catalyst for synthesis of 5-substituted 1H-tetrazoles from nitriles and sodium azide. Chin. J. Chem..

[101] Lang L, Zhou H, Xue M, Wang X, Xu Z (2013). Mesoporous ZnS hollow spheres-catalyzed synthesis of 5-substituted 1H-tetrazoles. Mater. Lett..

[102] Mani P, Singh AK, Awasthi SK (2014). AgNO_3_ catalyzed synthesis of 5-substituted-1H-tetrazole via [3+2] cycloaddition of nitriles and sodium azide. Tetrahedron Lett..

[103] Sreedhar B, Suresh Kumar A, Yada D (2011). CuFe_2_O_4_ nanoparticles: A magnetically recoverable and reusable catalyst for the synthesis of 5-substituted 1H-tetrazoles. Tetrahedron Lett..

[104] Agawane SM, Nagarkar JM (2012). Synthesis of 5-substituted 1H-tetrazoles using a nano ZnO/Co_3_O_4_ catalyst. Catal. Sci. Technol..

[105] Nikoorazm M, Ghorbani-Choghamaranai A, Khanmoradi M, Moradi P (2018). Synthesis and characterization of Cu(II)-Adenine-MCM-41 as stable and efficient mesoporous catalyst for the synthesis of 5-substituted 1H-tetrazoles and 1H-indazolo [1,2-b]phthalazine-triones. J. Porous Mater..

[106] Nikoorazm M, Rezaei Z, Tahmasbi B (2020). Two Schiff-base complexes of copper and zirconium oxide supported on mesoporous MCM-41 as an organic–inorganic hybrid catalysts in the chemo and homoselective oxidation of sulfides and synthesis of tetrazoles. J. Porous Mater..

[107] Kikhavani T, Moradi P, Mashari-Karir M, Naji J (2022). A new copper Schiff-base complex of 3,4-diaminobenzophenone stabilized on magnetic MCM-41 as a homoselective and reusable catalyst in the synthesis of tetrazoles and pyranopyrazoles. Appl. Organometal. Chem..

