# Supplementary material for: A new samarium complex of 1,3-bis(pyridin-3-ylmethyl)thiourea on boehmite nanoparticles as a practical and recyclable nanocatalyst for the selective synthesis of tetrazoles
Source: Sci Rep. 2023 Apr 11;13:5902. doi: 10.1038/s41598-023-33109-y (PMC10090185; doi:10.1038/s41598-023-33109-y)
Supplement: Supplementary file 1 — Supplementary Information. [file 41598_2023_33109_MOESM1_ESM.docx]

Supplementary Data

**A new samarium complex of 1,3-bis(pyridin-3-ylmethyl)thiourea on boehmite nanoparticles as a practical and recyclable nanocatalyst for the selective synthesis of tetrazoles**

**Parisa Moradi^^[[1]](#footnote-1)^*,a^, Tavan Kikhavani^*,b^, Yunes Abbasi Tyula^a^**

*^a^Department of Chemistry, Faculty of Science, Ilam University, P.O. Box 69315516, Ilam, Iran.*

*^b^Department of Chemical Engineering, Faculty of Engineering, Ilam University, Ilam, Iran.*

**Abstract:**

Boehmite is a natural and environmentally friendly compound. Herein boehmite nanoparticles were primarily synthesized and, then, their surface were modified via 3-choloropropyltrimtoxysilane (CPTMS). Afterwards, a new samarium complex was stabilized on the surface of the modified boehmite nanoparticles (Sm-bis(PYT)@boehmite). The obtained nanoparticles were characterized using thermogravimetric analysis (TGA), energy dispersive X-ray spectroscopy (EDS), Brunauer-Emmett-Teller (BET), wavelength dispersive X-ray spectroscopy (WDX), scanning electron microscope (SEM), Fourier transform infrared spectroscopy (FT-IR), Inductively coupled plasma mass spectrometry (ICP-MS), dynamic light scattering (DLS), and X-ray diffraction (XRD) pattern. Sm-bis(PYT)@boehmite was used as an environmentally friendly, efficient, and organic–inorganic hybrid nanocatalyst in the homoselective synthesis of tetrazoles in polyethylene glycol 400 (PEG-400) as a green solvent. Notably, Sm-bis(PYT)@boehmite is stable and has a heterogeneous nature. Thus, it can be reused for several runs without any re-activation.

**Keywords***:* Green solvent, Boehmite nanoparticles; Reusable catalyst; Environmentally friendly catalyst, Homoselective catalyst, Tetrazole.

**5-(2-chlorophenyl)-1H-tetrazole:** ^1^H NMR (400 MHz, DMSO): δ_H_= 7.81-7.78 (d, *J*= 12 Hz, 1H), 7.72-7.69 (d, *J*= 12 Hz, 1H), 7.64-7.60 (t, *J*= 8 Hz, 1H), 7.57-7.52 (t, *J*= 8 Hz, 1H) ppm.


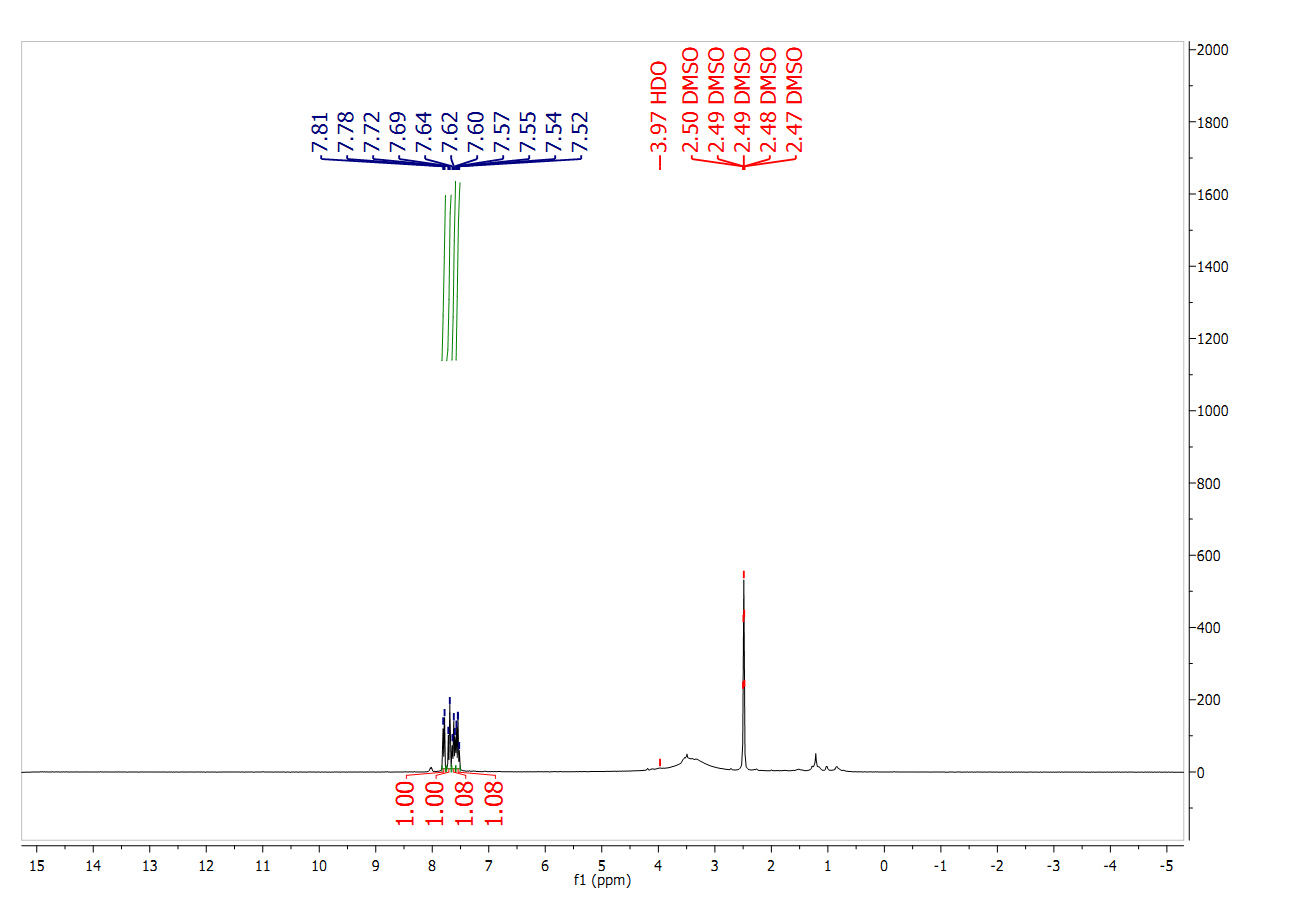


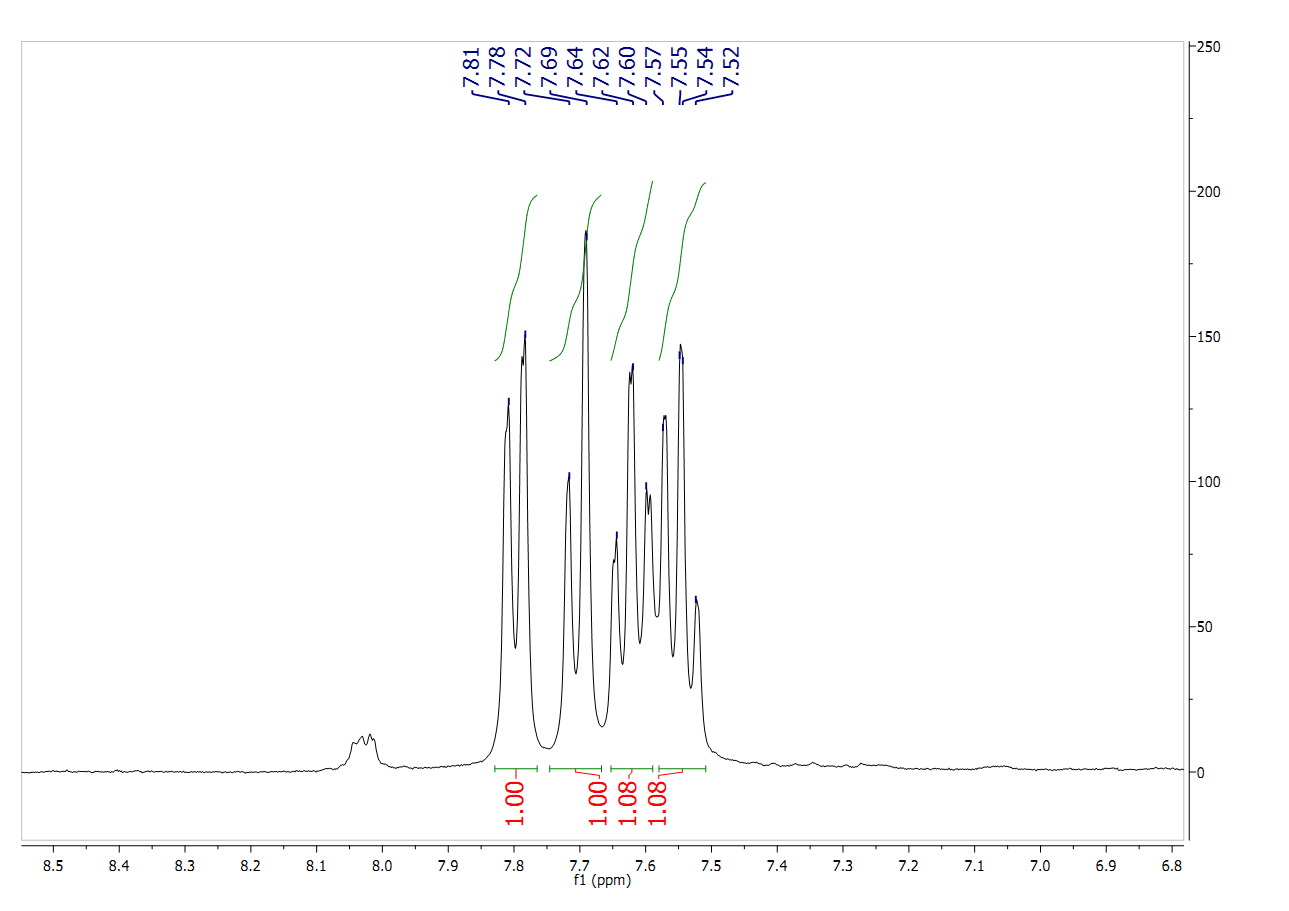


**Figure S1.** ^1^H NMR spectrum of synthesized 5-(2-chlorophenyl)-1H-tetrazole

**5-(4-nitrophenyl)-1H-tetrazole:** ^1^H NMR (400 MHz, DMSO): δ_H_= 16.55 (br, 1H), 8.46-8.43 (d, *J*= 12 Hz, 2H), 8.31-8.28 (d, *J*= 12 Hz, 2H) ppm.


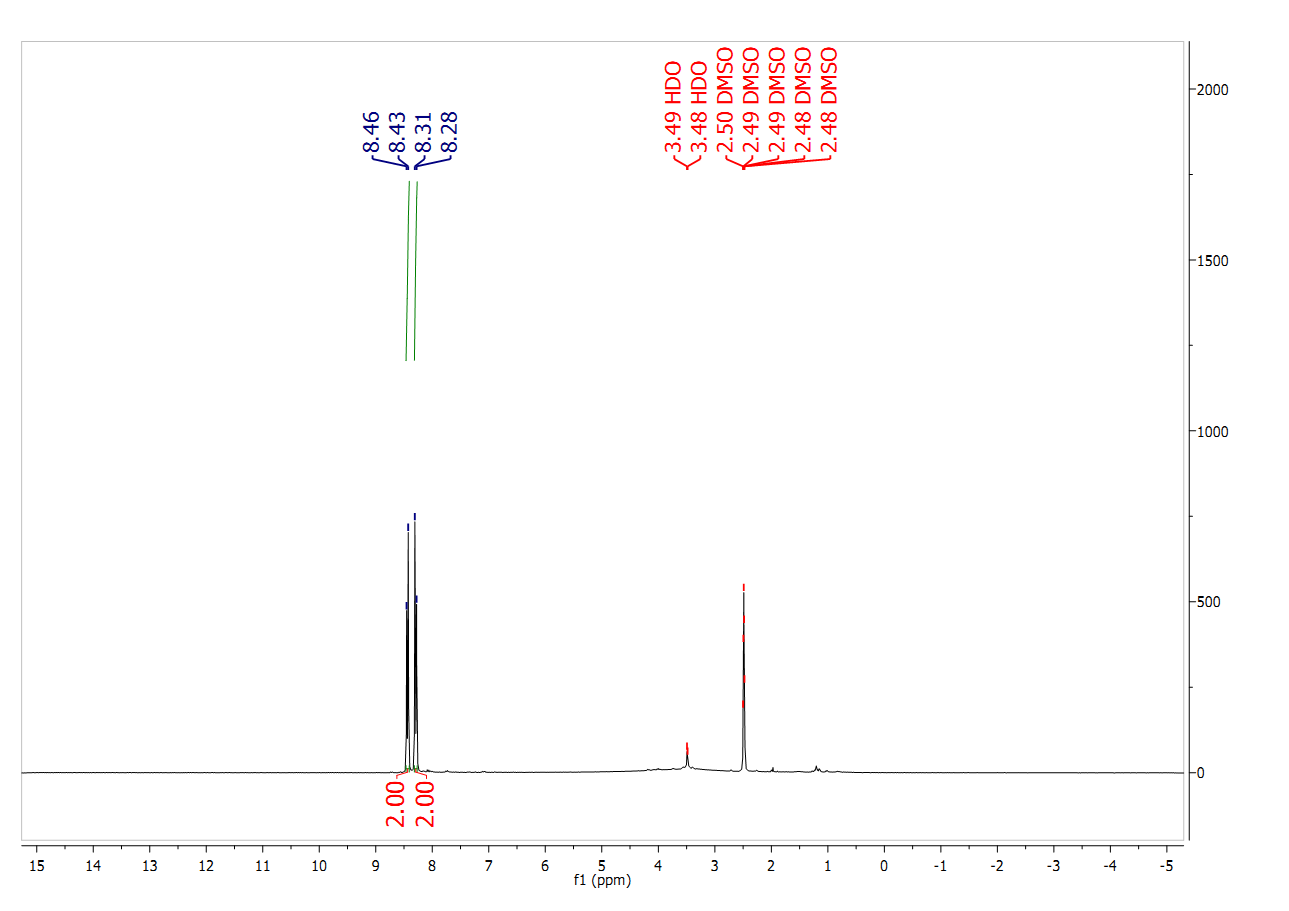

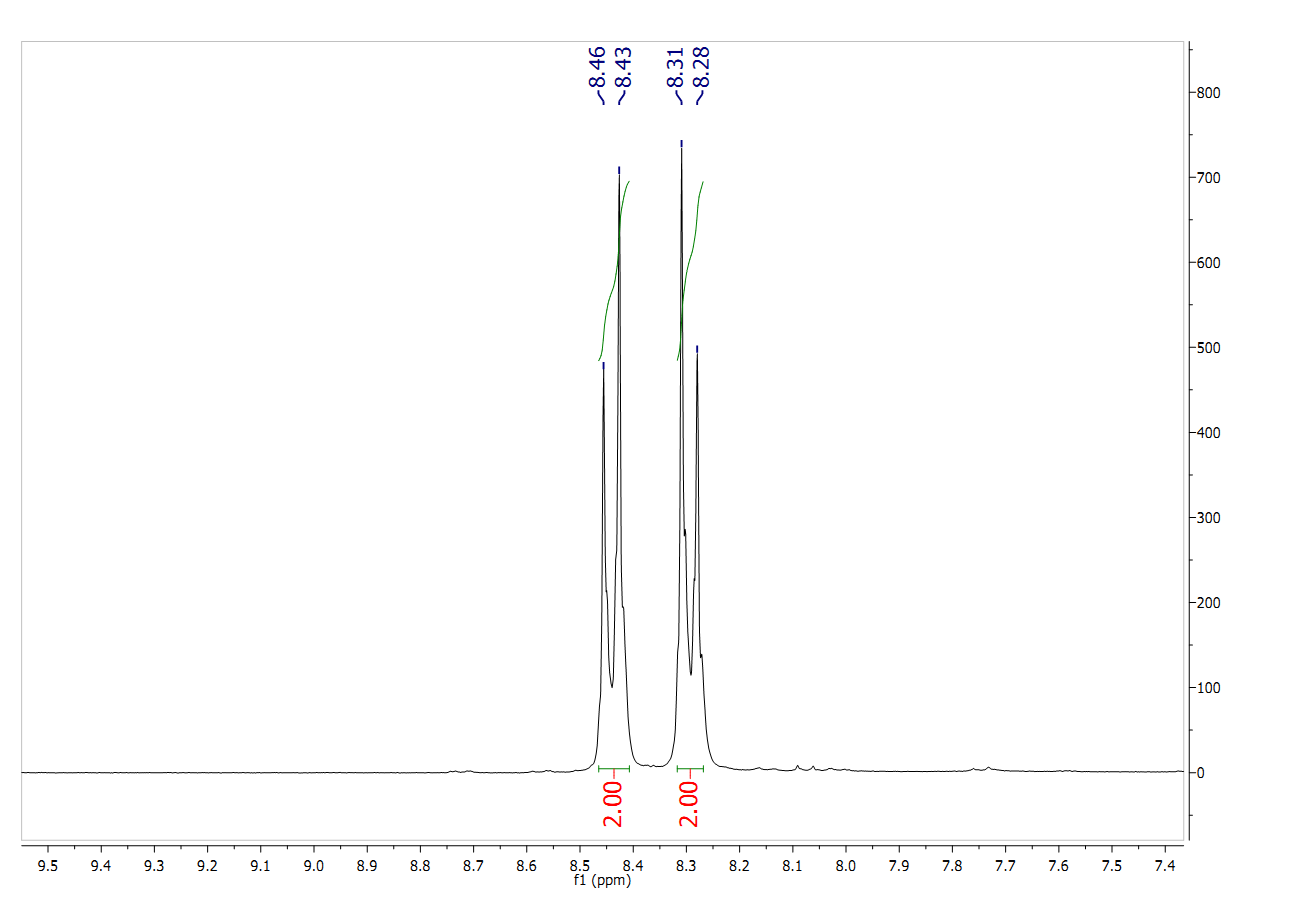


**Figure S2.** ^1^H NMR spectrum of synthesized 5-(4-nitrophenyl)-1H-tetrazole

1. * Address correspondence to P. Moradi, Department of Chemistry, Ilam University, P.O. Box 69315516, Ilam, Iran; Tel/Fax: +98 841 2227022; E-mail address: [p.moradi@ilam.ac.ir](mailto:p.moradi@ilam.ac.ir) and [parisam28@yahoo.com](mailto:parisam28@yahoo.com)

   * Address correspondence to T. Kikhavani, Department of Chemical Engineering, Faculty of Engineering, Ilam University, Ilam, Iran. E-mail address: [t.kikhavandi@ilam.ac.ir](mailto:t.kikhavandi@ilam.ac.ir)

   [↑](#footnote-ref-1)
